# Antimycobacterial Peptides: From Natural Product Discovery to AI Guided Design

**DOI:** 10.1021/acs.biochem.6c00132

**Published:** 2026-04-27

**Authors:** Diptomit Biswas, Scott H. Medina

**Affiliations:** † Department of Biomedical Engineering, 8082 Pennsylvania State University, University Park, Pennsylvania 16802-4400, United States; ‡ Molecular, Cellular, and Integrative Biosciences Program, Pennsylvania State University, University Park, Pennsylvania 16802-4400, United States; § Huck Institutes of the Life Sciences, Pennsylvania State University, University Park, Pennsylvania 16802-4400, United States

## Abstract

Mycobacterial pathogens remain major global health threats, exacerbated by both rapid acquisition of antibiotic resistance and the formidable drug diffusion barrier presented by the rigid mycomembrane. These challenges have renewed interest in antimycobacterial peptides (AMyPs), a diverse class of short amphiphilic sequences capable of rapidly killing both drug-sensitive and drug-resistant mycobacteria. Beyond their intrinsic potency, AMyPs can synergize with existing antibiotics and exhibit markedly slower resistance development relative to conventional small molecules. In this review, we synthesize recent advances spanning natural bioprospecting, mechanism-guided rational design, and chemical optimization strategies that have yielded increasingly potent and selective AMyP candidates. We further highlight the rapid emergence of artificial intelligence–driven discovery platforms, which leverage machine-learning models trained on curated, mycobacteria-specific data sets to predict and refine novel AMyPs with growing accuracy. Together, these technologic, biologic, and computational advances outline a rapidly expanding landscape for AMyP-based therapeutic development and establish a foundation for next-generation antimycobacterial drug design.

## Graphical abstract

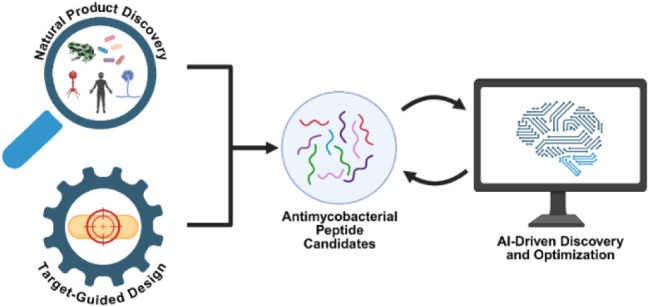

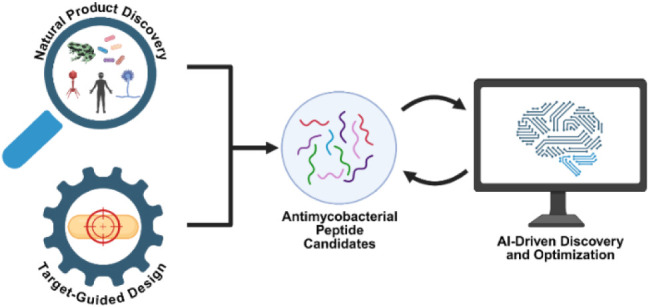

## 1 Introduction

The *Mycobacteriaceae* family encompasses a diverse array of bacteria occupying aquatic and terrestrial ecosystems.^1^ While the majority exist as benign environmental saprophytes, a small but consequential subset have evolved into highly successful human and animal pathogens. Foremost among these is *Mycobacterium tuberculosis* (*Mtb*), the etiological agent of tuberculosis (TB), a disease once known as the “White Plague” and still among the leading causes of death from a single infectious agent globally.^2^ In parallel, nontuberculous mycobacteria (NTMs), including *Mycobacteroides abscessus* and *Mycobacterium avium*, have emerged as increasingly prevalent opportunistic pathogens, with pulmonary infections rising at an estimated annual rate of 4% and exhibiting pronounced intrinsic antibiotic resistance.^3^ Beyond human disease, *Mycobacterium bovis*, the causative agent of bovine TB, continues to inflict substantial economic losses on the global livestock industry (>$3B annually), while maintaining a persistent zoonotic spillover threat.^4^ Together, these intersecting challenges underscore an urgent and ongoing need to expand and diversify the therapeutic strategies available to combat pathogenic mycobacteria.

The discovery of small molecule antibiotics, such as isoniazid and rifampicin, during the midtwentieth century transformed mycobacterial infections from a largely fatal disease into a curable condition.^5^ However, decades of selective pressure have driven the emergence and global dissemination of multidrug- and extensively drug-resistant (MDR/XDR) mycobacterial strains, compromising the effectiveness of existing therapies^6^ (Figure 1A). Consequently, current treatment regimens increasingly rely on prolonged, multidrug combinations that are costly and have significant side effects.^7^ Central to mycobacterial antibiotic tolerance is the unique composition of the mycomembrane, an outer barrier rich in waxy mycolipids that restricts the intracellular penetration of many small-molecule drugs^8^ (Figure 1B). Accordingly, there is growing interest in nontraditional antimycobacterial therapies capable of efficiently breaching this barrier to eliminate mycobacteria through direct cell lysis or biochemical inhibition of intracellular targets.^8,9^ Importantly, by compromising membrane integrity, such agents can potentiate the efficacy of current antibiotics, potentially resensitizing otherwise resistant strains to standard regimens.

**Figure 1:**
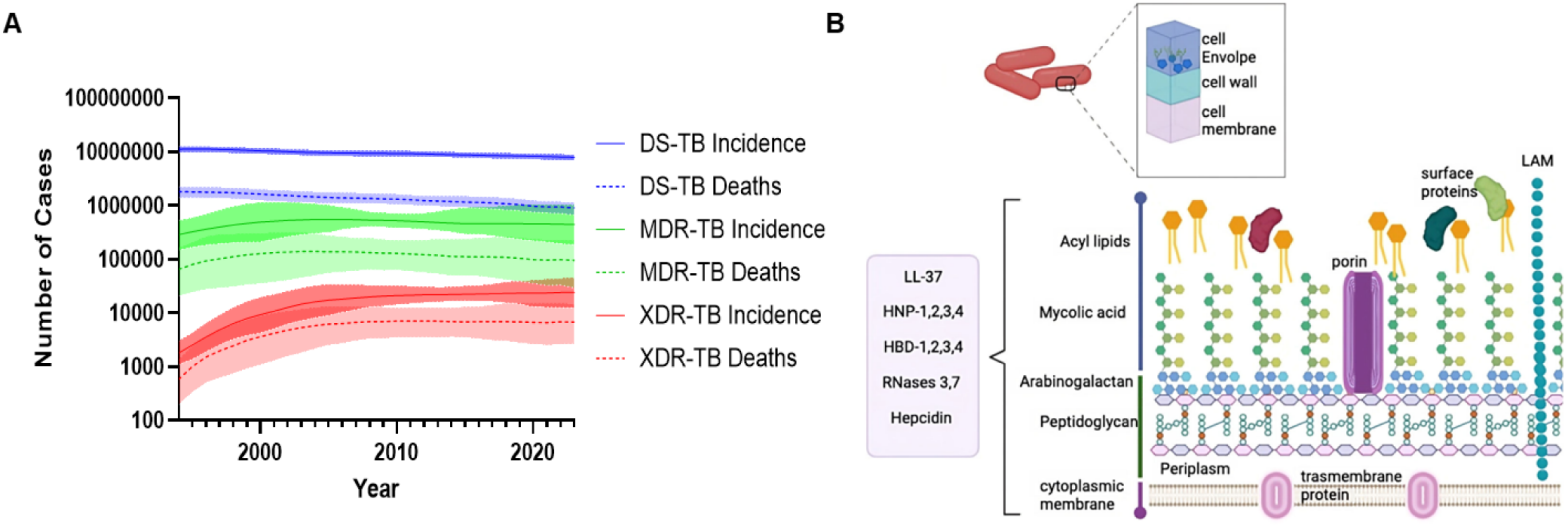
Recent *Mtb* incidence and mycomembrane composition. (A) Incidence data collected over the past three decades from the Global Burden of Disease Study 2023 (GBD 2023) by the Global Burden of Disease Collaborative Network, Seattle, United States: Institute for Health Metrics and Evaluation (IHME), 2024. Available from https://vizhub.healthdata.org/gbd-results/. Data was plotted using Graphpad. (B) Mycobacteria are characterized by a waxy outer membrane rich in mycolic acids, which can be permeabilized and destabilized by AMyPs. LAM = lipoarabinomannan. Figure is reproduced with permission from ref 9, copyright © 2023 Jacobo-Delgado, Rodríguez-Carlos, Serrano and Rivas-Santiago. Figure is licensed under CC BY 4.0, no changes were made to the graphic.

Among these emerging approaches, antimycobacterial peptides (AMyPs), a specialized subset of antimicrobial peptides exhibiting potent mycobactericidal activity, have emerged as leading candidates.^9,10^ Structurally diverse, these short (5–50 amino acids) amphiphilic sequences often occur naturally as linear α-helices or macrocyclic scaffolds and incorporate both proteinogenic and nonproteinogenic amino acids. Their amphiphilic character enables AMyPs to electrostatically engage the negatively charged mycomembrane, leading to rapid membrane destabilization and/or translocation into the cytosol to inhibit intracellular processes essential for mycobacterial survival and virulence.^10^ Crucially, both mechanisms impose a high barrier to resistance, as physical disruption requires an energetically costly remodeling of the cell envelope, while targeting conserved intracellular processes severely restricts the pathogen’s capacity for mutational escape.^11^ Recent advances in chemical sequence optimization, including enantiomerization, cyclization, and terminal modification, have begun to address the proteolytic and bioavailability barriers inherent to AMyP *in vivo* translation, transforming this class of compounds into clinically relevant therapeutic candidates.^12^

This review surveys recent advances in AMyP discovery and design over the past seven years, with a particular focus on emerging rational design methodologies and their structure-guided therapeutic optimization. Historically, AMyP discovery has relied on two distinct, but complementary, strategies. The first mirrors classical natural product discovery, involving the screening of diverse biological and chemical repositories derived from organisms that interact with mycobacteria, including hosts, ecologic competitors, and bacteriophages. The second approach, mechanism-guided rational design, integrates knowledge of essential mycobacterial pathways with insights from high-resolution structural techniques to engineer *de novo* highly potent candidates with defined targets. While powerful, both strategies demand substantial time and resource investments to yield clinically viable leads. Recently, artificial intelligence (AI)-driven discovery has emerged as a transformative solution to these bottlenecks, leveraging machine learning (ML) models to rapidly predict and optimize AMyP sequences *in silico*. Given the rapid proliferation of ML-based AMyP prediction tools in recent years, this review critically examines the recent literature and provides a conceptual framework to guide future AI-enabled antimycobacterial peptide discovery.

## 2 Natural AMyP Discovery

Bioprospecting of natural products has long been the foundation of antibiotic discovery, yielding many of the most effective TB drugs used today.^5^ Likewise, many potent classes of AMyPs have emerged from diverse environmental sources.^13^ Additionally, host defense peptides, antimicrobials expressed as part of innate immune responses against mycobacterial pathogens, have been identified as a rich repository for AMyP discovery.^14^ Indeed, through extensive evolutionary pressure, nature has generated a vast and chemically diverse repertoire of AMyPs across many domains of life.

### 2.1 Animal Sources

Animals, in particular, have coexisted with, and defended against, mycobacterial pathogens for millennia.^15^ The resulting arms race has accordingly produced a wide variety of potent AMyPs, ranging from host defense to encrypted peptides. The following section presents the latest updates in AMyP sequence discovery from animal biorepositories and discusses their efficacy and pharmacologic parameters relevant to utility as clinical therapeutics.

#### 2.1.1 Host Defense Peptides

The importance of host defense peptides (HDPs) in controlling mycobacterial infections is well established across the animal kingdom.^14^ Defensins are one such exemplary superfamily of cysteine-rich HDPs, serving as a cornerstone of innate immunity.^16^ In humans, these sequences are categorized into two major subfamilies, α- and β-defensins, distinguished primarily by the connectivity of their disulfide bonds (Figure 2). Prominent representatives include human Neutrophil Peptides (hNP 1–3) and human β-Defensins (hBD 1–3).^16^ Bioinformatic analysis has revealed >30 hBD-like sequences, although only a small subset have been experimentally characterized.^17^ A notable study by Rodriguez et al. evaluated hBD10, and a consensus peptide (hBD*consensus*) designed from the sequence alignment of all known β-defensins, against various pathogens, including *Mtb* ^17^ (Table 1). Both peptides exhibit strong antimycobacterial activity, with the improved performance of hBD*consensus* attributed to its greater cationic charge and structural stability. Incorporation of an N-terminal α-helix, commonly present in other defensins, was hypothesized to further enhance its potency.^17^

**Figure 2:**
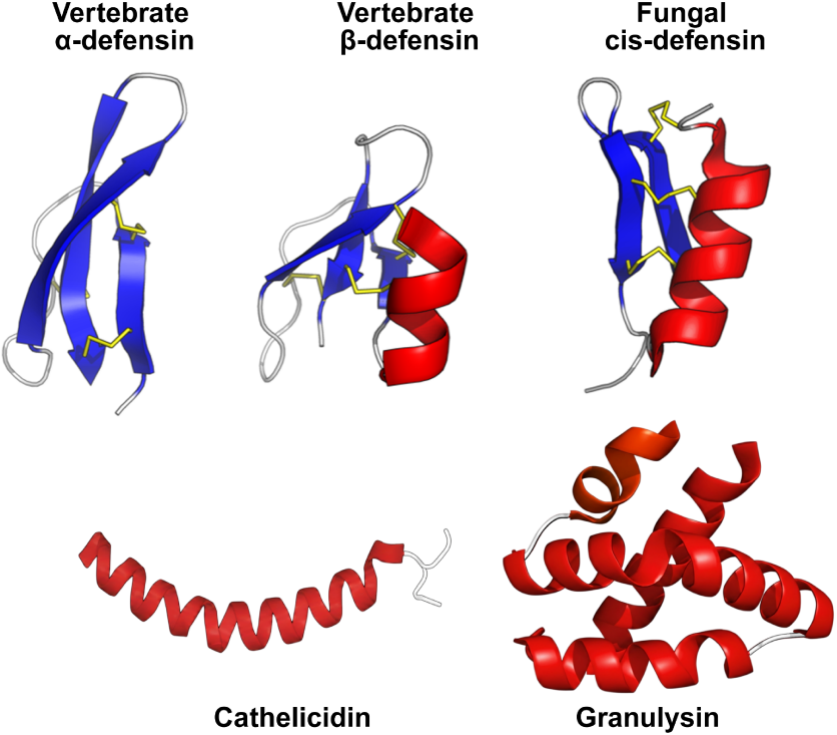
Host defense peptides possess diverse structural motifs. To ensure broad spectrum-antimicrobial activity, immune systems have weaponized HDPs with unique bioactive motifs, several of which show potent killing against *Mtb*. Vertebrate and fungal defensin structures adapted with permission from ref 195 copyright © 2008 Thomas Shafee. Figure is licensed under CC BY 4.0. Cathelicidin and Granulysin structures were rendered from PDB 2K6O and 1L9L in PyMOL and recolored to maintain visual consistency.

**Table 1:** Linear AMyP Sequences and Their Discovery Source, Molecular Target and Inhibitory Potency against Diverse Mycobacterial Species

Ref	Peptide name	Peptide sequence^a^	MIC^b^ (in μM)	Pathogen	Target/mechanism of action	Derived from
^17^	hBD10	RECRIGNGQCKNQCHENEIRIAYCIRPGTHCCLQQ	11.8	*Mtb* H37Rv	Membrane disruption	Human Gene DEFB110
 ^17^	hBD*consensus*	KKCWNGGRCRKKCKENEKPIGYCRNGKKCCVN	8.8	*Mtb* H37Rv	Membrane disruption	Synthetic
^18^	Pep-H	RRYGTCIYQGRLWAF	5.3	*Mtb* H37Rv	Membrane disruption	Human Neutrophil Peptide 1
 ^19^	Pep-B	ACPIFTKIQGTCYRG	12.1	*Mtb* H37Rv	Membrane disruption	Human β-Defensin 1
 ^24^	WBCATH	GLPWILLRWLFFRG	18	*Mtb* H37Ra	Membrane disruption	*Bubalus bubalis* Cathelicidin 4
 ^25^	Gran1	QRSVSNAATRVCRTGRSTWRDVCRNFMRR	1	*M. bovis* BCG	Membrane disruption	Human Granulysin
^26^	NKLF2	VCDKMKILRGVCKKIMRSFLRR	<2	*M. smegmatis*	Membrane disruption	Porcine NK-Lysin
^29^	B1CTcu5	LIAGLAANFLPQILCKIARKC	5.54	*Mtb* H37Rv	Membrane disruption	*Clinotarsus curtipes* skin
^32^	W3R6	VWRRWRRFWRR	25	*Mtb* H37Ra	Membrane disruption, Intracellular gDNA binding	*Rana chensinensis* skin
^32^	D-W3R6	vwrrwrrfwrr	25	*Mtb* H37Ra	Membrane disruption, Intracellular gDNA binding	*Rana chensinensis* skin
^32^	AC-D-W3R6	A*vwrrwrrfwrr	6.25	*Mtb* H37Ra	Membrane disruption, Intracellular gDNA binding	*Rana chensinensis* skin
^32^	BA-D-W3R6	B*-vwrrwrrfwrr	6.25	*Mtb* H37Ra	Membrane disruption, Intracellular gDNA binding	*Rana chensinensis* skin
^34^	Tachyplesin	KWCFRVCYRGICYRRCRGK	20	*M. smegmatis*	Membrane Disruption	*Tachypleus tridentatus* hemolymph
^34^	CyLoP-1	CRWRWKCCKK	10	*M. smegmatis*	Intracellular ROS generation, Membrane depolarization	*Crotalus durissus* toxin
^37^	O1_cal29b	RPKCCCVCGVVGRKCCSTWKDCHPVHLPCPSS	3.5	*Mtb* H37Rv	Unknown ion channel inhibition	*Californiconus californicus* toxin
^38^	I1_xm11a	GRCRGFREDCSQHRDCCGDLCCNGNTCVITVIACPKW	3	*Mtb* H37Rv	Unknown ion channel inhibition	*Conasprella ximenes* toxin
^42^	YQA-15	YQAMLLRIARIPMGL	3.5	*Mtb* H37Ra	Membrane disruption	Human Glutathione s-transferase theta
^42^	YRA-15	YRAMLLRIARIRMRL	1.6	*Mtb* H37Ra	Membrane disruption	Human Glutathione s-transferase theta
^42^	YWW-15	YWWMLLRIWRIWMRL	1.4	*Mtb* H37Ra	Membrane disruption	Human Glutathione s-transferase theta
^42^	RWW-15	RWWMLLRIWRIWMRL	1.4–2.8	*Mtb* H37Ra	Membrane disruption	Human Glutathione s-transferase theta
^44^	D-hLF 1-11	grrrrsvqwca	72.7	*Mtb* H37Rv	Membrane disruption	Human lactoferricin
^47^	Angie1	KAINTFIHGNKISIKAI	100	*Mtb* H37Rv	Membrane disruption	Human angiogenin
^48^	NapFab	TIGGIKGAKKIFGKALW	100	*Mtb* H37Rv	Membrane disruption	Human Napsin A
^61^	Lynronne 2D_all_	hlrrinklltriglyrhafg	10.7	*Mtb* H37Rv	Membrane disruption	Bovine gut microbiome
^61^	P15sD_all_	kfvrlkiycrdknkgrgisf	37.9	*Mtb* H37Rv	Membrane disruption	Bovine gut microbiome
^64^	NZ2114	GFGCNGPWNEDDLRCHNHCKSIKGYKGGYCAKGGFVCKC	6.1	*M. bovis* BCG	Inner membrane disruption	Fungal Plectasin
^66^	NZX	GFGCNGPWSEDDIQCHNHCKSIKGYKGGYCARGGFVCKCY	6.3	*Mtb* H37Rv	Inner membrane disruption, ACP, EF Tu, 60 kDa chaperonin 1	Fungal Plectasin
^86^	Synthetic PK34	PRVIETKVHGREVTGLARNVSEENVDRLAKRWIK	12.6	*Mtb* H37Rv	Membrane disruption	D29 mycobacteriophages
^87^	Recombinant PK34	PRVIETKVHGREVTGLARNVSEENVDRLAKRWIK	3.2	*Mtb* H37Rv	Membrane disruption	D29 mycobacteriophages
^89^	AK15	AKKKLSRWWLRWWVK	18.1	*Mtb* H37Rv	Membrane disruption	Che12 mycobacteriophages
^89^	Ak15-6	AVKKLLRWWSRWWKK	9.1	*Mtb* H37Rv	Membrane disruption	Che12 mycobacteriophages
^92^	MAD1	KRWHWWRRHWVVW	5	*Mtb* H37Rv	Membrane Disruption	*M. smegmatis* porin MspA
^95^	AKVUAM 1	YYYYEEKWW	>360	*Mtb* H37Rv	*Mtb* Cpn T—human macrophage CR1 interaction	Synthetic
^95^	AKVUAM 2	YYRRYHHHQ	>360	*Mtb* H37Rv	*Mtb* Cpn T—human macrophage Surfactant D interaction	Synthetic
^99^	IP1	KFLNRFWHWLQLKPGQPMY	99.27	*Mtb* H37Rv	Membrane disruption, Autophagy	Synthetic
^105^	V30-SP-8	S*LAAG*RHS*	25	*M. smegmatis*	*Mtb* VapC30 toxin	*Mtb* VapB30
 ^106^	V26-SP-8	R*AELAVLS*ELA	25	*M. smegmatis*	*Mtb* VapB26 antitoxin	*Mtb* VapC26
 ^116^	FGE inhibitor 1	SMMMC	>100	*M. smegmatis*	Formylglycine-generating enzyme	Synthetic
^116^	FGE inhibitor 2	SCGMM	>100	*M. smegmatis*	Formylglycine-generating enzyme	Synthetic
^165^	D-LAK120-A	kklalalakkwlalakklalalakk	50	*Mtb* H37Ra	Membrane disruption	Synthetic
^165^	D-LAK120-HP13	kkalahalkkwlpalkklahalakk	12.5	*Mtb* H37Ra	Membrane disruption	Synthetic
^182^	TAA-GS Peptide 2e	T*VO*LT*VO*L	1.5	*Mtb* H37Ra	Membrane disruption	*Brevibacillus brevis* Gramicidin S
^186^	ASU2001	QFNGrSkaAkVNFwrka	256	*M. abscessus*	NA	Synthetic
^186^	ASU2009	rYGlSkArkVNQFRkal	256	*M. abscessus*	NA	Synthetic
^186^	ASU2019	rVGPSAPHNlFrrkSal	256	*M. abscessus*	NA	Synthetic
^186^	ASU2056	QrwGlSlAPYkNFrrlS	32	*M. abscessus*	NA	Synthetic
^186^	ASU2059	YGrSArYNrrklGalSG	256	*M. abscessus*	NA	Synthetic
^186^	ASU2060	VGrwSArYNFrwrkSGl	8	*M. abscessus*	NA	Synthetic
^27^	NKLF2-M16C	VCDKMKILRGVCKKICRSFLRR	NA	NA	Membrane disruption, DNA gyrase, Emb C, RPS1 and ACP	Porcine NK-Lysin
^27^	NKLF2-M16I	VCDKMKILRGVCKKIIRSFLRR	NA	NA	Membrane disruption, DNA gyrase, Emb C, RPS1 and ACP	Porcine NK-Lysin
^96^	BFH	P*KLVFFWHSGTPH	NA	NA	Traps Mycobacteria	Synthetic
^101^	E9-α4	DEDREWEGTVGDGLG	NA	NA	MazF-mt1 and MazF-mt9 toxins	Synthetic
^101^	E1-α3	TLEDDYANAWQEWSAAG	NA	NA	MazF-mt1 toxins	Synthetic
^107^	RNAP inhibitor 1	EGKLYPVE	NA	NA	RNAP-CarD interaction	*Thermus thermophilus* TRCF protein
^107^	RNAP inhibitor 2	TGEIKSQT	NA	NA	RNAP-CarD interaction	*Mtb* RNA Polymerase β-subunit
^108^	Peptide 16	TPAQGMIFSVKVRTGITSVSVKAWLFWRMREDFQPDTD	NA	NA	Rho helicase	Bacteriophage P4 Psu
^108^	Peptide 33	TPAQFIFFRVKVRNWHTSVSVKAWLFWRMREDFQPDTD	NA	NA	Rho helicase	Bacteriophage P4 Psu
^112^	DBAASP17881	DDDY	NA	NA	Membrane disruption, Acetate Kinase inhibition	Synthetic
^117^	P578	FKARREQERMKKLGA	NA	NA	Protein Kinase B inhibition	Synthetic

a Abbreviations: Acetyl (A*), Butanoyl (B*), Bis-pentenylglycine (G*), (R)-7-octenylalanine (R*), (S)-4-pentenylalanine (S*), Tetrahydrofuran amino acid (T*), Ornithine (O*), Bis-pyrene (P*).

b Minimum Inhibitory Concentration (MIC) values reported in μg/mL have been converted to μM using reported/calculated peptide molecular masses.

Given the relatively large size of human defensin peptides (30–40 residues), recent efforts have focused on identifying shorter fragments using bioinformatic prediction tools such as iAMPred and CellPPD. Through this strategy, two 15-residue sequences, Pep-B and Pep-H, were derived from hBD-1 and hNP-1, respectively^18,19^ (Table 1). Although these truncated peptides displayed reduced potency toward virulent *Mtb*, they were significantly less pro-inflammatory and cytotoxic than the parent sequence. Notably, both molecules include a conserved γ-core motif, consisting of two antiparallel β-sheets connected by a hairpin loop, which has been identified as a key structural determinant of defensin antimicrobial activity.^20^

Cathelicidins represent another major superfamily of HDPs integral to innate immune defense.^14^ These amphipathic α-helical sequences eliminate pathogens by disrupting their outer membranes and recruiting immune cells, demonstrating notable activity against *Mtb* ^21^ (Figure 2). The human cathelicidin LL-37, for example, eliminates intracellular *Mtb* by inducing autophagy through a Vitamin D-dependent pathway.^22^ Interestingly, unlike humans and mice, which possess only one cathelicidin gene (LL-37), water buffaloes have seven distinct sequences.^23^ This expanded arsenal likely reflects an evolutionary adaptation to frequent environmental exposure to mycobacteria in swamp and floodplain habitats. Accordingly, water buffalo (*Bubalus bubalis*) represents a promising biorepository for AMyP discovery. Palacios et al. reported potent *in vitro* and *in vivo* anti-TB activity of WBCATH, a 14 residue C-terminal fragment of *B. bubalis* cathelicidin 4 ^24^ (Table 1). Follow up *in-silico* simulations suggest the peptide’s activity is due to its self-assembly into octameric pore-like structures within the mycomembrane.^24^

Additional cysteine-rich HDPs with notable antimycobacterial activity include granulysin and its porcine homologue, NK-lysin.^25^ These peptides adopt a saposin fold composed of five amphipathic helices stabilized by disulfide bonds (Figure 2). Upon appropriate physiologic stimulation, this structure undergoes conformational rearrangement to expose an internal helix–loop–helix motif capable of hydrophobic membrane engagement.^25^ Because full-length saposin peptides are large (∼9 kDa), costly to synthesize, and associated with off-target immune toxicity, research has largely focused on helix–loop–helix derived fragments. Two such fragments, Gran 1 (from granulysin) and NKLF2 (from NK-lysin), exhibit micromolar inhibitory activity against several mycobacterial species (Table 1).^25,26^ Despite similar potencies, these peptides operate via distinct mechanisms of action: Gran 1 disrupting membrane integrity, and NKLF2 predicted to inhibit key metabolic protein targets like DNA gyrase, arabinosyltransferase C (Emb C), ribosomal protein S1 (RPS1), and acyl carrier proteins (ACP).^27^ Further *in silico* optimization of NKLF2 variants to optimize antimycobacterial potency, pharmacokinetics, immunogenicity and target binding affinities, yielded two improved candidates, NKLF2-M16C and NKLF2-M16I, which remain to be experimentally validated.^27^

While these mammalian derived HDPs represent the fruits of a highly evolved and coordinated immune system, older taxa, such as amphibians and invertebrates that possess limited or absent adaptive immune responses, instead rely heavily on innate defenses to combat mycobacterial pathogens.^15,28^ For example, frogs secrete a diverse array of antimicrobial peptides from specialized glands in their skin,^28^ with several shown to possess selective antimycobacterial activity. Abraham et al. isolated B1CTcu5, a brevinin-1 variant from the Indian frog *Clinotarsus curtipes*, that selectively eliminates *Mtb* H37Rv via membrane disruption, while exhibiting little activity against *Mycolicibacterium smegmatis* (Table 1).^29^ Structure–activity relationship (SAR) studies indicates that the full-length sequence, including the C-terminal disulfide-bridged ring (also known as the Rana box), is essential for retention of bactericidal activity.^29^ Since this structural motif is also responsible for strong hemolytic activity,^30^ additional SAR studies are required to further optimize this sequence before clinical translation is warranted.

Another amphibian-derived antimicrobial peptide, chensinin-1, has been isolated from the frog species *Rana chensinensis*, and demonstrated to be active in killing *Mtb* H37Ra.^31,32^ While chensinin-1’s antimycobacterial potency is relatively modest, a truncated derivative, W3R6 and its proteolytically stable enantiomer D-W3R6, enriched in tryptophan and arginine residues, displayed significantly enhanced activity.^32^ The addition of short fatty-acyl chains (specifically acetyl and butanoyl) to D-W3R6 further improved its antimycobacterial efficacy by enhancing its mycomembrane permeabilization (Table 1). In addition to membrane disruption, W3R6 and its analogues translocate into the cell to bind mycobacterial genomic DNA, providing a dual mechanism of action that substantially limits the potential for resistance development.^32^

Finally, horseshoe crabs (*Tachypleus tridentatus*), ancient marine invertebrates, are armed with specialized immune cells, called hemocytes, that release antimicrobial tachyplesin peptides from intracellular granules upon infection^33^ (Table 1). These sequences have recently been shown to exhibit potent inhibitory and antibiofilm activity against multiple mycobacterial species,^34^ and operate through mechanisms that include membrane destabilization and inhibition of biosynthetic enzymes important to cell wall integrity.^33^

#### 2.1.2 Venom-Derived Peptides

Nature’s deployment of peptides extends beyond immune defenses to include offensive predatory strategies, in which HDPs have been evolutionarily co-opted as toxic venoms.^35^ Consequently, several potent antibacterial sequences, like melittin and scorpine, have been identified in venom mixtures, with a subset active toward mycobacteria.^36^ For instance, CyLoP-1 (cytoplasmic localizing peptide-1), a sequence derived from the South American rattlesnake (*Crotalus durissus*), possesses low micromolar inhibitory activity and antibiofilm effects toward various mycobacterial species. Mechanistic studies indicate that CyLoP-1 exerts its antimycobacterial activity through membrane depolarization and generation of reactive oxygen species^34^ (Table 1). Structure–activity analysis further revealed that disulfide bond formation within the peptide is critical for both its antimycobacterial potency and cellular uptake. Marine predators, particularly cone snails, represent another rich source of venom-associated peptides. Conotoxins, such as l1_xm11a (from *Conasprella ximenes*) and O1_cal29b (from *Californiconus californicus*), demonstrate low micromolar antimycobacterial activity when tested *in vitro* (Table 1), highlighting venom-derived peptides as a complementary and underexplored reservoir of AMyPs.^37,38^

#### 2.1.3 Encrypted Peptides

Distinct from the constitutive activity of HDPs and venoms, there exist a vast reservoir of sequences with antimicrobial activity that remain dormant within proteins until proteolytically released.^39^ These sequences, referred to as encrypted peptides, remain functionally silent until enzymatic degradation of the parent protein liberates bioactive peptide fragments, often triggered by inflammation or tissue damage.^40^ This mechanism allows a single host protein to serve multiple biological roles, while simultaneously providing a rapid, locally deployable antimicrobial response. A notable example is human glutathione s-transferase (GST)-theta, an enzyme responsible for detoxifying xenobiotics via glutathione conjugation, which contains several encrypted AMyP sequences that were released following incubation with human neutrophil elastase.^41^ Recently, these helical sequences were further modified to enhance their cationic and hydrophobic nature, greatly improving their interaction with the lipid-rich mycomembrane and leading to markedly enhanced antimycobacterial efficacy.^42^ Notably, incorporation of a tryptophan-rich C-terminus further increased potency, underscoring the importance of aromatic residues in AMyP membrane interactions (Table 1).

Lactoferrin, an iron-chelating glycoprotein found in milk and other secretions, represents another prolific source of encrypted AMyPs.^43^ Its positively charged N-terminal fragment, human lactoferricin (hLF), is primarily generated via partial hydrolysis in the stomach and plays an important role in the innate immunity of newborn infants. hLF and its shorter derivatives such as hLF(1–11) exhibit broad-spectrum antimicrobial activity, including potent inhibition of multiple mycobacterial species.^43,44^ Recently, Intorasoot et al. showed that the synthetic d-isomer of hLF(1–11) offers improved *in vitro* stability and a more favorable hemolytic profile relative to the natural L-chiral parent, while maintaining equivalent potency against *Mtb* H37Rv^44^ (Table 1). Camel milk, in particular, represents a rich source of lactoferrin-derived AMyPs. In a recent study, Kanawati et al. utilized *in silico* digestion of camel milk proteins to predict several antimicrobial sequences that inhibit the *Mtb* thymidylate kinase, an enzyme that catalyzes ATP-dependent phosphorylation en route to nucleotide biosynthesis.^45^ While these putative sequences have yet to be validated experimentally, they represent potential therapeutic scaffolds since *Mtb* lacks a compensatory pathway for this critical biofunction, making enzyme inhibition lethal to the pathogen.^46^ Additionally, its low homology with human analogues supports further development of these theoretical AMyP sequences.^46^

Blood serum and pulmonary fluids also harbor encrypted antimicrobial peptides that contribute toward infection resistance. Angiogenin, a 123-residue protein secreted by immune cells to promote angiogenesis, was recently identified as a serum-derived AMyP based on its potent inhibitory activity against extracellular *Mtb*. Additional computational analysis revealed that a 17-residue cytolytic sequence encrypted within angiogenin is responsible for this inhibition, which was further modified to form the synthetic AMyP Angie1.^47^ This sequence showed potent activity against *Mtb* and several ESKAPE pathogens, while being well tolerated by host macrophages (Table 1). Similarly, Beitzinger et al. identified a cryptic AMyP through *in vitro* screening of a peptide library derived from human bronchoalveolar lavage fluid. Mass spectrometry confirmed that this fragment was proteolytically obtained from Napsin A, an aspartic proteinase important for respiratory surfactant production. This sequence was further optimized *in silico* to improve its aqueous solubility, yielding the NapFab peptide, which demonstrated greater efficacy in disrupting the *Mtb* cell envelope (Table 1).^48^ While effective toward extracellular bacteria, both Angie1 and NapFab show limited activity toward intracellular persister bacilli due to poor proteolytic stability and inefficient cellular uptake. Therefore, both studies employed nanoparticulate delivery vehicles to effectively transport Angie1 and NapFab to the phagolysosomes of infected macrophages to eliminate resident *Mtb* bacilli.^47,48^

### 2.2 Bacterial and Fungal Sources

Ecologically crowded environments, like soil and marine ecosystems, host diverse bacterial and fungal species which are in constant competition with environmental mycobacteria.^49,50^ This sustained ecologic pressure has catalyzed the evolution of structurally diverse peptides designed to suppress and kill mycobacterial competitors. In this section, we discuss the mechanisms and efficacy of recently discovered AMyPs derived from these environments, and distinguish them by their ribosome-dependent or ribosome-independent synthetic origins.

#### 2.2.1 Ribosomal Peptides

Bacteriocins are ribosomally synthesized antimicrobial peptides that are produced by bacteria and can be subdivided into multiple structural classes.^51^ Among these, class I bacteriocins, belonging to the superfamily of Ribosomally synthesized and Post-translationally Modified Peptides (RiPPs), have yielded several exciting AMyP leads in recent years.^51^ Initially synthesized from canonical amino acids, RiPPs undergo extensive post-translational modification, including macrocyclization and cross-linking, to adopt a final active form.^51^ Among these, lasso peptides, characterized by a threaded lariat topology that confers exceptional stability, have emerged as particularly attractive scaffolds.^52^ Triculamin, a recently identified member of this class, exhibits remarkable stability and potent inhibitory activity specifically against mycobacteria^53^ (Table 2). Mechanistic studies of the closely related peptide lariocidin suggest that triculamin exerts its activity through inhibition of the bacterial ribosome.^54^ Similarly, lanthipeptides, defined by their characteristic thioether cross-links, represent another prominent family of RiPPs.^51^ Solabiomycins A and B, two recent entrants to this family, exhibit potent inhibition of virulent *Mtb*, which is strictly dependent on the presence of a sulfoxide-stabilized rigid structure within the peptide sequence^55,56^ (Table 2). Besides these structurally defined leads, another novel antimycobacterial RiPP, Pantocin wh-1, has been recently discovered from *Pantoea dispersa* W18 following nutrient stress^57^ (Table 2). In contrast, class II bacteriocins, which remain largely unmodified, are frequently identified in gastrointestinal microbiota.^58^ Notable examples include the bovine gut-derived peptides Lynronne-1, -2, -3 and P15s.^59,60^ While these native sequences showed limited efficacy against *Mtb*, their synthetic d-enantiomers successfully cleared intracellular mycobacteria from infected macrophages without causing significant off-target toxicity^61^ (Table 1).

**Table 2:** Cyclic AMyP Sequences and Their Discovery Source, Molecular Target and Inhibitory Potency against Diverse Mycobacterial Species

Ref	Peptide name	Peptide sequence^a^	MIC^b^ (in μM)	Pathogen	Target/mechanism of action	Derived from
^53^	Triculamin	cyc(SKKSKPGD)GIRGKGVRG	0.073	*M. smegmatis*	Ribosome inhibition	*Streptomyces*
^55^	Solabiomycin A	1*GG^2*IA^^GGA^	3.49	*Mtb* H37Rv	Membrane disruption	*Streptomyces lydicus*
^55^	Solabiomycin B	1*GG^2*VA^^GGA^	3.55	*Mtb* H37Rv	Membrane disruption	*Streptomyces lydicus*
^57^	Pantocin wh-1	cyc(VMWCYVFGYGFNCAVW)	3.89	*Mtb* H37Ra	Membrane disruption,ROS production	*Pantoea dispersa* W18
 ^69^	Wollamide B	cyc(WLlVNo*)	2.97	*Mtb* H37Rv	Unknown	*Streptomyces*
^69^	Wollamide B1	cyc(WLlVNr)	2.97	*Mtb* H37Rv	Unknown	*Streptomyces*
^69^	Wollamide B2	cyc(WLlVNk)	0.99	*Mtb* H37Rv	Unknown	*Streptomyces*
^71^	Atratumycin	cyc(SWnF(β-o)PvGlyT)-c(2-m)	14.6	*Mtb* H37Rv	Unknown	*Streptomyces atratus* SCSIO ZH16
 ^72^	Atrovimycin	cyc(3*T*SlF(β-h)St*s4*Vl)	1.9	*Mtb* H37Rv	Unknown	*Streptomyces*
^75^	Atratumycin B	cyc(5*TSWnF(β-o)PvGly)	2.3	*Mtb* H37Rv	Unknown	*Streptomyces* (mutant)
^76^	Coprisamide C	6*-cacyc(slV7*D(β-m))V	90	*Mtb* mc^2^ 6230	Unknown	*Micromonospora* UTJ3
 ^77^	Icosalide 1a	cyc(lS8*h*SL8*p*)	7.04	*Mtb* H37Ra RFP	Membrane disruption	*Burkholderia gladioli*
^77^	Icosalide 2	cyc(lS8*t*SL8*-m)	21.01	*Mtb* H37Ra RFP	Membrane disruption	*Burkholderia gladioli*
^77^	Icosalide 4	cyc(lS8*n*SL8*h*)	3.57	*Mtb* H37Ra RFP	Membrane disruption	*Burkholderia gladioli*
^77^	Icosalide 13	cyc(lS9*h*SL9*p*)	7.62	*Mtb* H37Ra RFP	Membrane disruption	*Burkholderia gladioli*
^82^	Asperversiamide A	cyc(swSavFv)	>100	*Mtb* H37Ra	Membrane disruption	*Aspergillus versicolor*
^82^	Asperversiamide B	cyc(swAavFv)	100	*Mtb* H37Rv	Membrane disruption	*Aspergillus versicolor*
^82^	Asperversiamide C	cyc(awSavFv)	100	*Mtb* H37Ra	Membrane disruption	*Aspergillus versicolor*
^81^	Asperheptatides A	cyc(swSGvFv)	100	*Mtb* H37Ra	Membrane disruption	*Aspergillus versicolor*
^81^	Asperheptatides B	cyc(awAavFv)	100	*Mtb* H37Ra	Membrane disruption	*Aspergillus versicolor*
^81^	Asperversiamide A-9	cyc(wSavFvs)-c(2,3-f)	12.5	*Mtb* H37Ra	Membrane disruption	*Aspergillus versicolor*
^81^	Asperversiamide A-13	cyc(wSavFvs)-c(4-o)	12.5	*Mtb* H37Ra	Membrane disruption	*Aspergillus versicolor*
^81^	Asperversiamide A-23	cyc(wSavFvs)-c(2-m)	12.5	*Mtb* H37Ra	Membrane disruption	*Aspergillus versicolor*
^81^	Asperversiamide A-24	cyc(wSavFvs)-c(4-m)	12.5	*Mtb* H37Ra	Membrane disruption	*Aspergillus versicolor*
^84^	Versicomycin	cyc(w(10*)SavFvs)	70	*Mtb* H37Ra	Membrane disruption	*Aspergillus versicolor*
^84^	CHNQD-02353	cyc(sw(2–11*)SavFv)	0.25	*Mtb* H37Ra	Membrane disruption	*Aspergillus versicolor*
^111^	Cyclohexyl-Griselimycin	N***N*VP(γ-m)cyc(N*TLP(γ-m)LN*VP(γ-ch)N*lG)	0.5	*M. abscessus* (ATCC 19977)	DnaN Sliding Clamp	*Streptomyces*
^113^	Lysocin E	cyc(12*TrSGN*FLrEqwIT)	0.309	*Mtb* H37Rv	Menaquinone (Vitamin K2)	*Lysobacter* RH2180-5
 ^121^	Callyaerins A	cyc(IOVILPPL)-d*PIFG	3.13	*Mtb* H37Rv	*Mtb* protein Rv2113	*Callyspongia aerizusa*
^121^	Callyaerins B	cyc(IOIILPPL)-d*PII	3.13	*Mtb* H37Rv	*Mtb* protein Rv2113	*Callyspongia aerizusa*
^128^	Cyclomarin A	cyc(AF(β-o)VN*L14*W(β-h,-e)N*L(δ-h))	0.1	*Mtb* H37Rv	ClpC1	*Streptomyces*
^129^	Rufomycin 4	cyc(W(−e)N*LY(3-n)AN*L(−p)L13*)	0.02	*Mtb* H37Rv	ClpC1	*Streptomyces*
^138^	Homo-BacPROTAC6	cyc(VN*LVF(β-m)AN*L(δ-h)W)-l*cyc(WVN*LVF(β-m)AN*L(δ-h))	0.34	*Mtb* H37Rv	ClpC1	Synthetic
^138^	Homo-BacPROTAC7	cyc(N*LVF(β-m)AN*L(δ-h)W(N*)A)-l*cyc(AN*LVF(β-m)AN*L(δ-h)W(N*))	0.26	*Mtb* H37Rv	ClpC1, ClpC2	Synthetic
^142^	Rufomycin 21	cyc(W*N*LY(3-n)AN*L(−p)L13*)	0.0098	*Mtb* H37Rv	ClpC1	*Streptomyces atratus* SCSIO ZH16 ΔilaR
^142^	Ilamycin J	cyc(W**N*LY(3-n)AN*L(−p)L13*)	0.0096	*Mtb* H37Rv	ClpC1	*Streptomyces atratus* SCSIO ZH16 ΔilaR
^145^	Cyclomarin A′	cyc(AF(β-m)VN*L14*W(β-h,-e)N*L(δ-h))	0.03	*Mtb* H37Rv	ClpC1	Synthetic
^142^	Cyclomarin C	cyc(AF(β-o)VN*L14*(p)W(β-h)N*L(δ-h))	0.25	*Mtb* Erdman WT	ClpC1	*Streptomyces*
^147^	Desoxycyclomarin C	cyc(AF(β-o)VN*L14*(p)WN*L(δ-h))	0.93	*Mtb* Erdman WT	ClpC1	Synthetic
^147^	Nitro-Desoxycyclomarin C (16e)	cyc(AF(β-o)VN*L14*(p)WN*L(δ-h))	0.13	*Mtb* Erdman WT	ClpC1	Synthetic
^149^	Metamarin	cyc(W*(β-h)N*L(δ-h)VF(β-o)VN*LV)	0.157	*Mtb* H37Rv	ClpC1	Soil
^150^	Ilamycin derivative 26	cyc(W(N*)N*LY(3-n)AN*L(−p)L13*)	0.05	*Mtb* H37Ra	ClpC1	Synthetic
^151^	Rufomycin-2024–58	cyc(W(−e)N*LY(3-n)AN*15*L13*)	0.0085	*Mtb* H37Rv	ClpC1	*Streptomyces*
^152^	Ecumicin	N**VVIcyc(TN*TVN*LVN*VN*W(4-o)VF(β-h)V)	0.16	*Mtb* H37Rv	ClpC1	*Nonomuraea*
^158^	OMS A	N*VVcyc(TN*TVN*LVN*VN*W(4-o)VF(β-h)V)	0.057	*Mtb* H37Rv	ClpC1	*Streptomyces*
^158^	OMS B	N*VIcyc(TN*TVN*LVN*VN*W(4-o)VF(β-h)V)	0.117	*Mtb* H37Rv	ClpC1	*Streptomyces*
^160^	dehydroxy-OMS A	N*VVcyc(TN*TVN*LVN*VN*W(4-o)VFV)	0.0049	*Mtb* mc^2^ 6230	ClpC1	*Streptomyces*
^160^	demethoxy-OMS A	N*VVcyc(TN*TVN*LVN*VN*WVF(β-h)V)	0.0402	*Mtb* mc^2^ 6230	ClpC1	*Streptomyces*
^159^	desmethoxydeoxy-OMS A (5)	N*VVcyc(TN*TVN*LVN*VN*WVFV)	0.25	*Mtb* mc^2^ 6230	ClpC1	Synthetic
^159^	desmethoxydeoxy-OMS A (10)	N*VVcyc(TN*TVN*LVN*LN*WVFV)	0.25	*Mtb* mc^2^ 6230	ClpC1	Synthetic
^159^	desmethoxydeoxy-OMS A (15)	N*VVcyc(TN*TVN*LVN*FN*WVFV)	0.125	*Mtb* mc^2^ 6230	ClpC1	Synthetic
^163^	Ecu★ 4	N*VVcyc(TN*TVN*LVN*AN*WVFV)	0.5	*Mtb* mc^2^ 6230	ClpC1	Synthetic
^164^	R_4_W_4_	cyc(RRRRWWWW)	2.91	*Mtb* Erdman WT	Membrane disruption	Synthetic
^185^	Streptomycobactin	N*VIITVLLVR(α-h)AVLAcyc(TVRIQR(α-h)V)	0.0133	*Mtb* H37Rv	Unknown	*Streptomyces*
^185^	Kitamycobactin	cyc(GFGRIKAD)QLVGRLIP	0.0346	*Mtb* H37Rv	ClpC1	*Kitasatospora*
^187^	Thurinsyn	cyc(y*O*rIRL)	6.96	*Mtb* H37Rv	Membrane depolarization	*Bacillus thuringiensis*

a Abbreviations: Cinnamoyl (−c), cyclohexyl (−ch), diaminoacrylic acid linker (-d*), N-(2,3-epoxy-3-methylbutyl) (−e), fluoro (−f), hydroxy (−h), Triazole-based linker (-l*), methyl (−m), nitro (−n), methoxy (−o), 4-methyl-5-hydroxy-2-piperidinone ring (−p), N-methyl (N*), N,N-dimethyl (N**), N-acetyl (N***), l/d-Ornithine (O*/o*), l/d-allothreonine (T*/t*), N-prenyl-Trp (W*), N-(3-Methyl-3-hydroxybut-1-enyl)-Trp (W**), Hydroxyproline (O), trityl (t*), pentyl (p*), heptyl (h*), nonyl (n*), myristoyl (y*), 13-guanidinotridecanoyl (1*), dehydrobutyrine (2*), 2-(1,2-diol-3-pentenyl)­cinnamoyl (3*), d-Hydroxyphenylaglycine (4*), N-2-(10,12-tetradecadienyl)­cinnamoyl (5*), 3-[2-(6-methyl-1,3-heptenyl)­phenyl]-2-propenoic acid (6*), 2,3-diaminopropanoic acid (7*), β-Hydroxy acid (8*), β-Amino acid (9*), 2-(2,4-dimethoxyphenyl) (10*), 4-(2-naphthyl)­phenyl (11*), 3-hydroxy-5-methylhexanoic acid (12*), 2-Amino-4-Hexenoic Acid (13*), 2-amino-3,5-dimethylhex-4-enoic acid (14*) and 5-hydroxy-5-methyl-2-pyrrolidinone (15*), sulfoxide bridges(^). Macrocyclic structure is indicated by cyc­().

b MIC values reported in μg/mL have been converted to μM using reported/calculated peptide molecular masses.

Fungi also contribute to the known array of ribosomally synthesized AMyPs, but through a distinct structural framework. Unlike mammalian α- and β-defensins (as discussed in Section A1), fungal defensins adopt a *cis*-defensin architecture featuring a cysteine-stabilized α-helix/β-sheet motif that confers exceptional proteolytic stability^62^ (Figure 2). Plectasin, a prototypical fungal cis-defensin, has served as the basis for several potent and proteolytically stable antimycobacterial derivatives.^62^ Two such analogues, NZX and NZ2114, showed improved phagolysosomal delivery and enhanced clearance of intracellular TB pathogens relative to the parent peptide^63,64^ (Table 1). Mechanistic studies demonstrated that both sequences exert their antimycobacterial activity through inner mycomembrane destabilization.^65^ Additionally, NZX also targets intracellular chaperonins and acyl carrier proteins to disrupt protein folding and mycolic acid biosynthesis.^66^ The ability to be recombinantly expressed in fungi, along with their demonstrated efficacy *in vivo*, make plectasin-derived peptides compelling candidates for further therapeutic development.^63,64^

#### 2.2.2 Nonribosomal Peptides

As a converse to ribosomal synthesis, many bacteria and fungi produce bioactive peptides through nonribosomal peptide synthetase (NRPS) pathways.^51^ These massive modular enzyme complexes assemble peptides independently of mRNA templates, with sequence identity dictated by the organization of enzymatic domains encoded within dedicated biosynthetic gene clusters (Figure 3). This mechanism permits direct incorporation of nonproteinogenic residues, extensive stereochemical variation, and formation of complex macrocyclic architectures that are inaccessible to ribosomal synthetic mechanisms.^51^

**Figure 3:**
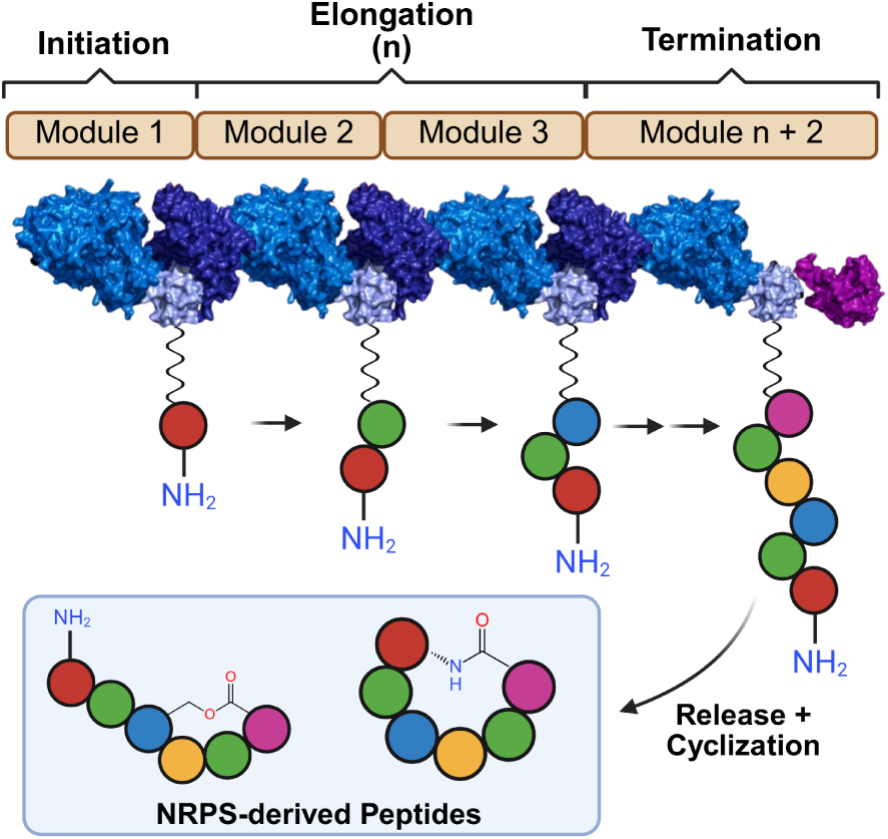
Modular architecture and biosynthetic logic of NRPS enzymes. NRPS complexes function as enzymatic assembly lines where discrete modules catalyze the initiation, elongation, and termination of the peptide chain. Following chain release, the product often undergoes cyclization, transforming linear intermediates into complex, bioactive secondary metabolites. Figure adapted with permission from ref 196 and modified with BioRender.com, copyright © 2024 the authors, some rights reserved; exclusive licensee from the American Association for the Advancement of Science.

Among bacteria, actinomycetes (especially *Streptomyces*) are renowned as prolific sources of bioactive NRPS-derived peptides that inhibit mycobacteria, such as capreomycin and rufomycin, with potencies spanning the low-micromolar to the nanomolar range.^67^ In many cases, this specificity stems from the targeted inhibition of essential enzymatic systems governing mycobacterial survival and virulence (as detailed below in Sections B2 and B3), although for many peptide families the molecular target remains undefined. Wollamides, cyclic hexapeptides isolated from a soil *Streptomyces* species, exemplify this class and show submicromolar inhibitory potency against *M. bovis*.^68^ These peptides share a conserved sequence comprising tryptophan, leucine, d-leucine, valine/isoleucine, asparagine and d-ornithine residues. Comprehensive SAR analysis of 36 synthetic analogues revealed strict residue- and stereochemistry-dependent activity, with substitutions at positions I and II eliminating activity, III–V had minimal to no effect, and modifications at position VI often enhancing activity.^69^ Two optimized analogues, Wollamide B1 and Wollamide B2, where d-Orn is substituted for other basic d-chiral residues, led to potent submicromolar activity toward *Mtb* H37Rv (Table 2). Stereochemistry was found to be particularly important, as replacing d-Orn with l-Lys led to a complete loss of activity. Additional studies showed that only Wollamide B1 effectively reduced intracellular *Mtb* populations in macrophages and is active against nutrient-starved dormant *Mtb* persisters.^69^ A later study reported that Wollamide B1 also exhibits synergism with frontline TB drugs, including against MDR and XDR strains.^70^

Additional NRPS-derived AMyPs, but with yet undefined mechanisms of action, include Atratumycin and Atrovimycin, both representing cyclic peptides isolated from *Streptomyces atratus* and *Streptomyces atrovirens* LQ13, respectively.^71,72^ These peptides fall within a class known as depsipeptides, characterized by their incorporation of both amide and ester bonds in their macrocycle, and both possess unique cinnamic acid groups that improve their hydrophobically driven interactions with mycomembranes.^73,74^ Atrovimycin demonstrated superior *in vitro* and *in vivo* efficacy against mycobacterial pathogens relative to Atratumycin (Table 2). Since the incorporation of cinnamoyl acid moieties in cyclodepsipeptides is extremely rare in nature, both studies uncovered novel biosynthetic pathways and enzymes important to AMyP production.^71,72^ Using these biosynthetic elements, Yang et al. designed Atratumycin analogues with unsaturated side chains of different lengths on their cinnamic acid groups, and demonstrated that increasing length of the unsaturated cinnamoyl motif correlates with increased antimycobacterial potency^75^ (Table 2). Coprisamide C, another cinnamic acid-containing cyclic depsipeptide produced by *Micromonospora* UTJ3, exhibits modest antimycobacterial activity against *Mtb* mc^2^ 6230 ^76^ (Table 2).

Lipophilicity-driven antimycobacterial effects are also employed by icosalides. These NRPS-derived cyclic depsipeptides possess two lipid chains and exhibit low micromolar antimycobacterial activity, with potency found to correlate with the peptide’s acyl chain length^77^ (Table 2). Although initially attributed to fungi, genome mining later revealed that icosalides are produced by *Burkholderia gladioli*, an endosymbiont of fungi.^78,79^ SAR investigation showed that the reactive ester bonds in these depsipeptides can be replaced with stable amide bonds without a significant loss in antimycobacterial potency.^77^ Furthermore, NMR experiments revealed that icosalides adopt an antiparallel β-sheet conformation that aligns the acyl chains on one face and the polar groups on the opposite face, creating a facially segregated amphipathic character that enables surfactant-like membrane disruption.

*Aspergillus versicolor*, a marine fungal species, represents a particularly rich source of NRPS-derived bioactive peptides.^80^ Recent breakthroughs in LC-MS/MS-dependent molecular networking have yielded several new sequences which show potent activity against mycobacteria.^81^ For example, two closely related cycloheptapeptides, Asperversiamides^82^ and Asperheptatides,^81^ were found to be potent inhibitors of the competitive marine mycobacterial pathogen *M. marinum*, with asperversiamide A demonstrating more potent bioactivity. However, both peptides showed limited efficacy against *Mtb* (Table 2). SAR studies revealed that the identity and stereochemical configuration of residues in position II and IV are of vital importance to their bioactivity, suggesting that these residues can be further optimized for enhanced mycobacterial potency.^81,82^ In fact, incorporation of modified cinnamic acid groups at these two positions in asperversiamide A led to a 8-fold increase in the peptide’s mycobacterial inhibitory activity^81^ (Table 2). Further discovery efforts yielded asperversiamide A derivatives with unusual tryptophan modifications, including the pyrroloindoline modified peptide asperpyrroindotide A^83^ and C2 aryl-substituted versicomycin sequence^84^ (Table 2). The former modification eliminated the antimycobacterial activity of the peptide, while the latter significantly enhanced its activity against *Mtb* H37Ra through increased macrocyclic rigidity. Interestingly, the C2 substitution observed in versicomycin is the result of a natural biosynthetic C–C coupling strategy that has not previously been reported.^84^ These dramatic substitution-dependent changes prompted a more expansive SAR study of different aryl substituted tryptophans. Notably, inclusion of a biaryl group, leading to spatial separation between the tryptophan indole ring and neighboring phenylalanine benzene ring, produced the analogue CHNQD-02353 with nanomolar antitubercular efficacy^84^ (Table 2). Additional *in vitro* studies demonstrated CHNQD-02353 had several promising pharmacologic attributes, including rapid and myco-specific killing, biofilm inhibition, low host toxicity and synergism with multiple frontline TB antibiotics. *In vivo* studies in murine infection models with *M. marinum*further validated its therapeutic potential, although additional optimization is required to address limitations in gastric stability and proteolytic half-life.^84^

### 2.3 Mycobacteriophages

With over 14,000 reported isolates, mycobacteriophages represent a vast and largely untapped source for AMyP discovery.^85^ Through prolonged coevolution with their mycobacterial hosts, these viruses have developed highly specialized lytic proteins that selectively recognize and engage mycomembrane lipids, thereby providing a rich genomic source of membrane-active peptide templates.^85^ PK34, derived from the D29 mycobacteriophage, was the first reported of its class. PK34 disrupts mycobacterial membranes via interactions with Trehalose-6,6′-dimycolate (TDM), a key glycolipid in the mycobacterial cell wall^86^ (Table 1). Beyond its structural role, TDM is a potent immunomodulatory molecule that drives granuloma formation and amplifies proinflammatory cytokine production via MAPK and PKB signaling pathways.^86^ By selectively binding TDM, PK34 attenuates these inflammatory responses, resulting in improved immunopathological outcomes compared to conventional antibiotics, such as rifampicin. In murine TB models, PK34 reduced pulmonary bacterial load to a level comparable to rifampicin drug controls, while being superior in minimizing inflammation-mediated lung damage.^86^ However, despite its therapeutic promise, PK34 is relatively large and, consequently, difficult to chemically synthesize. Recent efforts have begun to address this limitation by recombinantly producing the peptide in *Escherichia coli* and engineered murine mesenchymal stem cell expression systems.^87,88^ Interestingly, recombinant PK34 showed greater potency than chemically synthesized analogues, an effect attributed to its enhanced folding in the presence of the SUMO fusion expression tag^87^ (Table 1). Alphafold-based structural modeling of PK34–TDM complexes further revealed that a flexible loop region anchors the peptide to the trehalose moiety via hydrogen bonding, stabilizing the interaction and enabling α-helix–mediated membrane disruption.^87^

Building upon this success, another TDM-binding peptide, AK15, was identified from an uncharacterized protein encoded in the genome of the Che12 mycobacteriophage.^89^ This cationic amphiphile is less than half the length of PK34 and possesses an α-helical secondary structure important for membrane lysis. Rational rearrangement of AK15 residues to enhance facial amphiphilicity yielded a more potent analogue, AK15–6 (Table 1). Both AK15 and AK15–6 significantly reduced pulmonary bacterial loads in infected mice, while concurrently mitigating TDM-associated inflammatory tissue damage.

## 3 Target-Guided AMyP Design

While natural products offer an array of potent antimycobacterials, their broad and pleiotropic mechanisms of action are often insufficiently selective to discriminate host cells and mycobacteria, resulting in significant dose limiting toxicities in humans.^90^ The convergence of advanced *in silico* modeling with an increasingly detailed understanding of mycobacterial biology has enabled a more rational approach: Mechanism-guided design (Figure 4). By explicitly linking molecular architecture to defined biological function, this strategy addresses both specificity and pharmacokinetic limitations through rational, structure-guided sequence development.^91^ We have previously demonstrated the utility of this approach through the porin-mimetic design of MAD1 (Mycomembrane-Associated Disruption-1 sequence), an AMyP derived from the mycobacterial proteome itself^92^ (Table 1). Hypothesizing that the transmembrane domains of mycobacterial outer membrane proteins are evolutionarily optimized to integrate within the unique mycolic acid-rich envelope, we utilized the mycobacterial porin MspA as a structural template. Through *de novo* engineering, MAD1 was designed to mimic specific transmembrane features of MspA to facilitate its selective insertion and assembly within mycomembranes, yielding a potent and selective AMyP.^92^ While the mycomembrane is an attractive target for antimycobacterial sequence design, the field has largely favored the development of AMyPs that prevent host cell infection or specifically target metabolic vulnerabilities and virulence mechanisms in mycobacteria.

**Figure 4:**
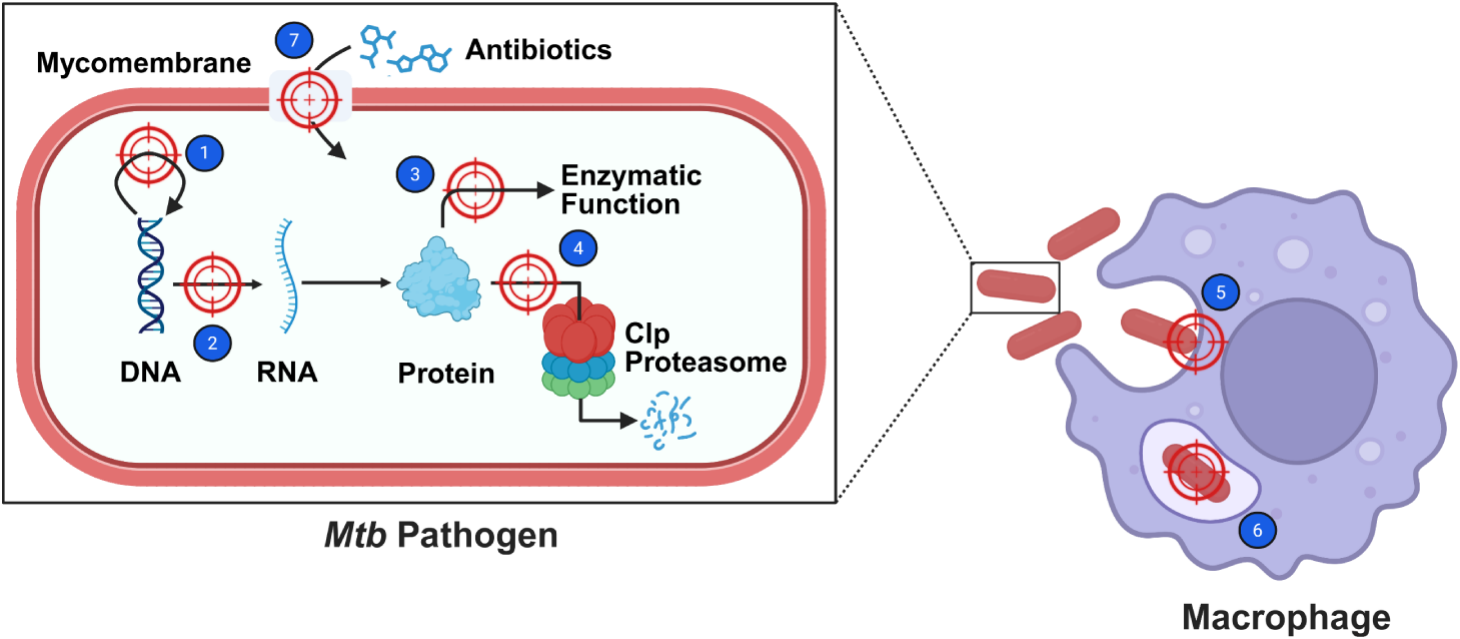
Overview of AMyP mechanistic targets. (1) Replication, (2) Transcription, (3) Enzymes involved in pathogen survival and virulence, (4) Protein quality control, (5) Host cell invasion (6) Mycobacterial persistence within host cells and (7) Mycomembrane integrity. Figure created with BioRender.com.

### 3.1 Targeting Invasion and Persistence within Host Cells

Inhibiting mycobacterial invasion of host cells, particularly macrophages, is a promising and underexplored strategy for AMyP discovery. Utilizing molecular docking, Simsek et al. identified two peptides predicted to inhibit intracellular bacilli uptake into macrophages by blocking interactions between the *Mtb* surface protein Cpn T^93^ and macrophage CR1 surface receptor^94^ (Table 1). The lead candidate, AKVUAM-2, although itself not bactericidal, reduced intracellular invasion of *M. smegmatis* into THP1 human macrophages by 12.5% relative to controls.^95^ This targeted approach of blocking cell–cell interactions is complemented by recent *de novo* peptide design efforts to create indirect inhibitors that physically entrap mycobacteria to prevent their interactions with host cells. For example, the BFH anti-TB peptide was rationally engineered to incorporate three functional domains that promote its self-assembly into oligomeric peptide nanoparticles that organize at the surface of mycopathogens^96^ (Table 1). This sequence incorporated the self-assembling bis-pyrene motif, in combination with the fibril inducing KLVFF aggregation sequence, to initiate particle oligomerization, and was targeted to mycobacteria via surface display of the targeting domain WHSGTPH. Cellular studies confirmed BFH nanoparticles specifically bind to mycobacterial pathogens like *Mtb* and *M. bovis*, while avoiding nonmycobacteria, to trigger the peptide’s subsequent assembly into stable β-sheet nanofibers.^96^ The emergent network of entangled peptide fibrils ultimately traps the bacilli and prevents their interaction with coincubated macrophages. Follow-up studies are currently underway to explore the potential of this system to trap and kill *Mtb*. Importantly, *Mtb*’s ability to persist within macrophage phagolysosomes, a cellular niche that limits its exposure to antibiotics, is critical for its survival and disease persistence.^97^ Previous studies have demonstrated that cell-penetrating peptides (CPPs) such as LL-37 can eliminate intracellular *Mtb* by inducing autophagy in infected macrophages.^22^ Plaza et al. recently designed a synthetic CPP, IP-1, which features a *S. cerevisiae*–derived α-pheromone sequence modified at the N-terminus with an antibacterial warhead capable of lysing mycobacteria^98^ (Table 1). Notably, this peptide was able to induce autophagy in infected macrophages by binding to and sequestering intracellular ATP, which effectively cleared *Mtb* persisters without killing the host cells.^99^

Another potent strategy to reduce mycobacterial persisters involves the specific targeting of toxin-antitoxin (TA) systems using AMyPs. These prokaryote-exclusive features are essential for bacterial survival in hostile environments and typically consist of a proteolytically stable toxin protein paired with an unstable antitoxin partner.^100^ Under normal growth conditions, the antitoxin inhibits the toxin either through translational inhibition (type I), direct binding (type II/III) or other mechanisms (types IV–VIII). However, this inhibitory activity is disrupted during cellular stress, for example during host invasion and infection, due to reduced expression and degradation of the antitoxin counterpart.^100^ The released toxin disrupts bacterial DNA replication and protein synthesis, thereby inducing dormancy that enables pathogens like mycobacteria to persist within the host cell. *Mtb* possess a large and diverse array (>80) of these systems, the largest families of which, MazEF and VapBC, represent valuable targets for AMyP discovery and design.^100^ Chen et al. utilized crystallographic techniques to identify two sequences, E1-α3 and E9-α4, that functionally inhibit the *Mtb* toxins MazF-mt1 and MazF-mt9 by mimicking the binding of their cognate antitoxin MazE^101^ (Table 1). As the MazEF TA system is important for *Mtb* survival during antibiotic induced stress,^102^ these inhibitors may be potent combinatorial agents that can enhance the efficacy of current TB antibiotics when dosed together. Conversely, prolonged activation of *Mtb* toxins like VapC can also induce cell death by cleaving tRNAs essential for translation to cause irreversible damage.^103^ Lee et al. employed this strategy by designing AMyPs that can block the interaction of VapB antitoxin with their cognate toxin to prevent the formation of the inactive VapBC complex.^104^ Follow-up med-chem studies created chemically stapled analogues that lock the peptide candidates into an α-helical conformation important for cellular permeability, thus improving their bactericidal activity toward *M. smegmatis*.^105,106^ Two promising antimycobacterial leads, the VapC30 toxin binding peptide V30-SP-8, and VapB26 antitoxin binding sequence V26-SP-8, were subsequently generated, with future *in vivo* studies required to validate antimycobacterial efficacy in animal models (Table 1).

### 3.2 Targeting Gene Expression, Metabolism and Virulence

The essential role of RNA polymerase (RNAP) in bacterial gene transcription has made it an attractive target for antibiotic discovery,^5^ and more recently peptide-based mycobacterial inhibitors. Kaur et al. identified two sequences that inhibit interactions between the transcriptional regulator CarD and the *Mtb* RNAP β-subunit RpoB, causing instability in the RNAP open promoter complex and transcriptional failure^107^ (Table 1). Importantly, the highly conserved nature of this RNAP interface across various clinical MDR/XDR *Mtb* strains suggests that these rationally designed candidates have the potential to combat drug-resistant mycobacterial infections.^107^ In other work, Ghosh et al. designed bacterial transcription inhibitors that modulate Rho dynamics, an RNA/DNA helicase that releases RNAP from its template DNA to terminate transcription.^108^ The resultant stabilization of Rho-RNA-DNA complexes causes transcription stalling that eventually leads to mycobacterial cell death. Phenotypic screening of a peptide library prepared via random mutagenesis yielded two Rho inhibitor leads that were validated for mycobacterial lethality following expression in *M. smegmatis* and *M. bovis* BCG^108^ (Table 1). However, these peptides are large (38 residues) and therefore slow to penetrate across the thick mycolic acid membrane, indicating additional med-chem optimization may be necessary before clinical translation.

Peptide inhibitors of DNA polymerase have also remained attractive AMyP scaffolds, with the most widely studied family being the griselimycins. These NRPS-derived peptides, isolated from *Streptomyces* species, specifically target a hydrophobic cleft on the mycobacterial sliding clamp DnaN, which serves to complex DNA polymerase and DNA strands during replication.^109^ Consequently, griselimycins have been shown to inhibit the proliferation of both drug-sensitive and drug-resistant *Mtb* strains. Yet, despite their significant therapeutic promise, these cyclic depsipeptides have largely been abandoned for development due to their poor oral bioavailability.^110^ Recent outcomes from SAR studies, however, have renewed interest in this class of compounds through the identification of analogues with improved pharmacologic parameters. In particular, a proline-modified cyclohexyl analog of griselimycin showed efficacy against *Mtb* and *M. abscessus* *in vitro*, and yielded encouraging pharmacokinetic results in TB mouse models^109,111^ (Table 2).

Other efforts have focused on designing inhibitors that target metabolism and bioenergetic pathways in mycobacteria. For example, Subramaniyan et al. utilized *in silico* screening of the antimicrobial sequence databases APD3 and DBAASP to identify five candidates that bind and inhibit the active site of the AckA enzyme,^112^ a key metabolic regulator that catalyzes the reversible conversion of acetyl phosphate and ADP into acetate and ATP. Follow up molecular dynamics simulations led to the prioritization of the lead sequence DBAASP17881 as a candidate for follow up *in vitro* and *in vivo* validation (Table 1). Lysocin E has similarly been shown to disrupt cellular respiration by targeting menaquinone (Vitamin K2), an essential membrane cofactor in the mycobacterial electron transport chain^113^ (Table 2). This NRPS-derived cyclic depsipeptide, featuring a short lipid tail, was isolated from the soil bacterium *Lysobacter* RH2180–5 and originally found to display antibacterial activity against Methicillin resistant *Staphylococcus aureus* (MRSA).^114^ However, unlike facultative anaerobes like MRSA, which possess compensatory bioenergetic pathways during oxygen deprivation, *Mtb* is uniquely susceptible to Lysocin E as it lacks alternative energy generating mechanisms and is solely dependent on aerobic respiration, even under hypoxic conditions.^115^ While this dependency ensures rapid sterilization of both replicating and persister *Mtb* by Lysocin E, efficacy of the peptide remains poor against intracellular bacilli due to its limited intracellular uptake.^113^

Finally, enzymes essential for the survival and virulence of mycobacterial pathogens have also been the focus of peptide-based drug design. Asiimwe et al. used *in silico* docking simulations, combined with *in vitro* bioactivity assays, to identify two novel pentapeptides that possess micromolar inhibitory activity toward sulfatase enzymes responsible for glycosaminoglycan processing during mycobacterial tissue invasion^116^ (Table 1). Similar methods of virtual screening were employed by Deka et al. to identify inhibitors that interact with the ATP-binding hinge domain of Protein Kinase B (PknB).^117^ PknB is a transmembrane signaling receptor that acts as a serine/threonine kinase and plays a vital role in cell division and induction of hypoxia-induced dormancy.^118^ Five initial lead candidates, selected based on their PknB binding affinity from *in silico* simulations, were studied and the lead candidate P578 was selected for follow up *in vitro* studies (Table 1). Complementing these *in silico* screening strategies, natural product screening has also yielded promising candidates targeting mycobacterial survival mechanisms. Callyaerins A and B, for example, are NRPS-derived semicyclic peptides isolated from the marine sponge *Callyspongia aerizusa* that exhibit potent inhibition of *Mtb* and *M. bovis* pathogens by binding to the *Mtb* specific membrane protein Rv2113 ^119–121^ (Table 2). Although the exact function of Rv2113 is unknown, the hydrophobic binding of callyaerin peptides to this protein causes dysregulation of critical pathways involved in lipid biosynthesis and DNA repair, leading to *Mtb* growth inhibition.^121^

### 3.3 Targeting the Caseinolytic Proteasomal System

Over the past two decades, caseinolytic protease (Clp), an ATP-dependent serine protease that degrades damaged or misfolded proteins, has garnered widespread recognition as a metabolic vulnerability in bacteria, around which novel antibacterial strategies can be developed.^122^ Mycobacteria, in particular, are uniquely susceptible to Clp inhibition as they are almost exclusively dependent on this system for protein quality control and lack alternative proteolytic pathways commonly employed by other bacteria.^123^ The Clp complex consists of two distinct functional units: the heterotetradecameric proteolytic barrel, ClpP1P2, and an associated AAA+ ATPase unfoldase, the most well studied of which being ClpC1.^124^ Functioning as a regulatory chaperone, the ClpC1 hexamer binds to the disordered termini of substrate proteins and, in an ATP dependent mechanism, mechanically unfolds the protein and threads the polypeptide chain into the ClpP1P2 chamber for degradation (Figure 5A, B). Driven by evolutionary competition, other bacteria (predominantly *Streptomyces*) have developed potent ClpC1 peptide inhibitors that can be structurally classified into two families, cyclic heptapeptides and cyclic depsipeptides.^67^ While other Clp inhibitors exist, such as lassomycin and ADEPs, their potency is generally inferior to the NRPS-derived families mentioned above.^124^ Therefore, the following section focuses exclusively on the mechanism of action of cyclic heptapeptides and depsipeptides, with emphasis on recent SAR investigations.

**Figure 5:**
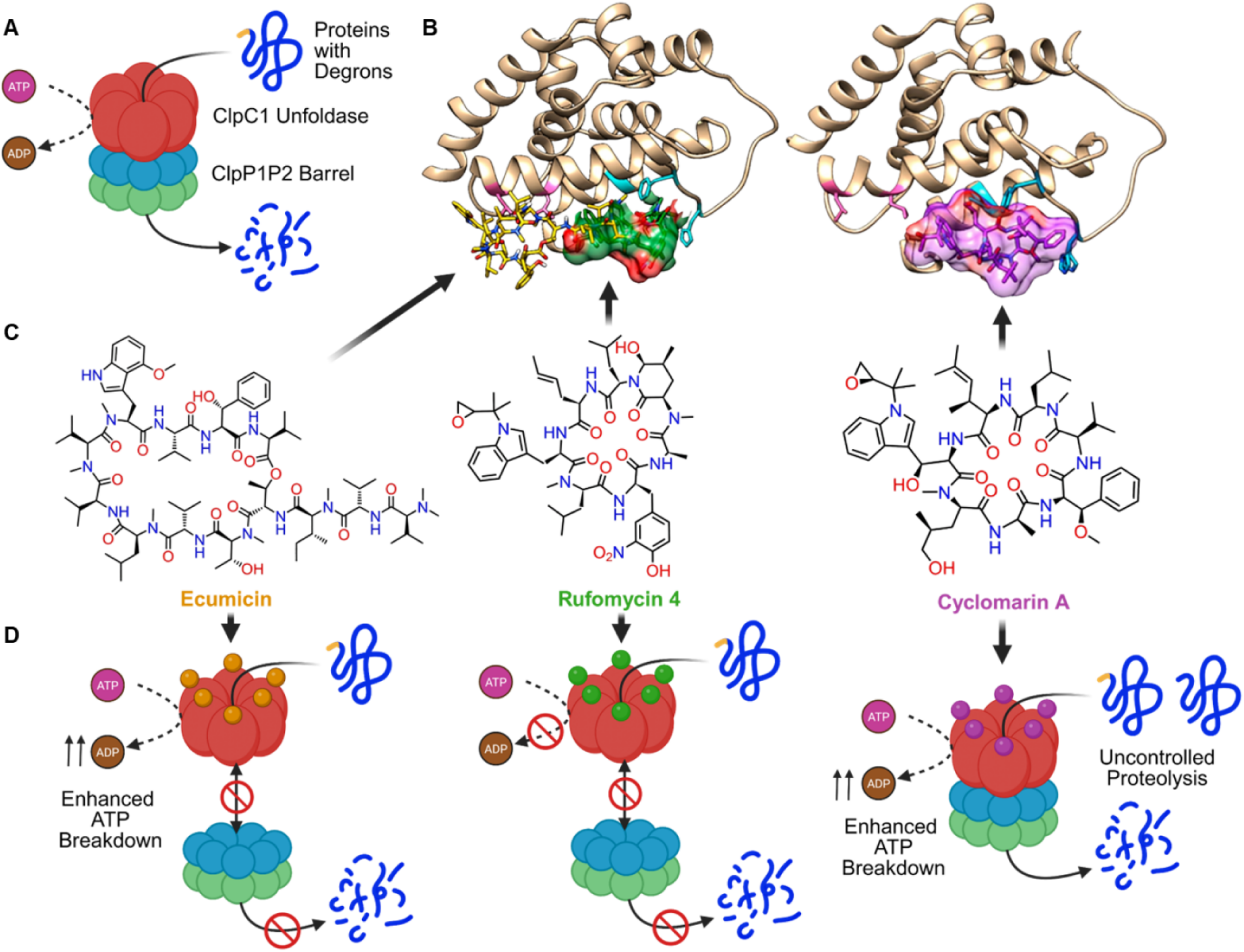
Normal and dysfunctional states of the Clp proteolytic system. (A) Under normal conditions, misfolded proteins with exposed degron sequences (yellow) interact with ClpC1 ATPase and are threaded into the ClpP1P2 barrel for degradation. (B) X-ray crystal structures of ClpC1 bound to cyclic peptides. (C) Chemical structures of ClpC1 inhibitors ecumicin, rufomycin 4 and cyclomarin. (D) Various mechanisms of action employed by each drug, all of which eliminate mycobacteria. Figure panels A and D were created with BioRender.com. Panels B and C were adapted with permission from ref 132, copyright © 2019 American Chemical Society.

#### 3.3.1 Cyclic Heptapeptides

ClpC1-targeting cyclic heptapeptides comprise two closely related classes, rufomycins (also known as ilamycins) and cyclomarins, isolated from different *Streptomyces* species in 1962 and 1999, respectively^125,126^ (Figure 5C). Although >30 natural rufomycin congeners have now been identified, rufomycin 4 and its diastereomers, rufomycin 5–7, constitute the major products extracted from fermentation broths.^127^ In contrast, the cyclomarin class is smaller, containing only four known natural congeners, with cyclomarin A and C serving as the primary subjects of investigation.^126^ Although the antimycobacterial nature of rufomycins was reported decades earlier, ClpC1 inhibition, and the corresponding modulation of proteasomal degradation, was only discovered in 2019, shortly after the same activity was identified for cyclomarins.^128,129^ Follow up bacteriological studies found that both rufomycin 4 and cyclomarin A inhibited the growth of sensitive and drug-resistant *Mtb* strains with nanomolar potency, and were effective in clearing intracellular dormant bacilli^128,129^ (Table 2). Furthermore, these agents demonstrated bactericidal efficacy against nontuberculous pathogens like *M. abscessus*.^129,130^ X-ray crystallography and cryo-electron microscopy studies showed that both inhibitors bind to the same hydrophobic ridge present in the N-terminal domain of ClpC1 and destabilize its decameric resting state^131–133^ (Figure 5B). Consequently, cyclomarin A promotes the assembly of hyperactive ClpC1 supercomplexes that lead to dysregulated proteolysis and cell death.^134,135^ In contrast, rufomycins do not alter ClpC1 activity, and instead eliminate *Mtb* cells by blocking ClpC1 interaction with the ClpP1P2 protease, thereby inhibiting essential proteolytic activity needed for survival^132^ (Figure 5D). However, mycobacteria have been shown to evolve compensatory mechanisms of resistance toward rufomycins and cyclomarins by expressing alternative unfoldases, like ClpX, and decoy proteins, like ClpC2.^136,137^ To bypass this, Hoi et al. designed a bivalent cyclomarin inhibitor named homo-BacPROTAC that binds to both ClpC1/ClpC2 proteins and the ClpP1P2 protease, subsequently promoting broad proteasomal disruption that is lethal to mycobacterial pathogens^138^ (Table 2).

Most rufomycin and cyclomarin congeners have three conserved standard residues, alanine, leucine and valine, as well as different presentations of four rare nonproteinogenic residues, which have prompted interest in SAR-based scaffold optimization. Initial efforts focused on the epoxide moiety of the prenylated tryptophan in both inhibitors, a residue that in cyclomarin A carries an additional β-hydroxy group. This specific focus was the result of mass spectrometry studies demonstrating that the epoxide forms a thioether linkage with ClpC1’s N-terminal methionine, implying that this group may be essential for bioactivity.^132^ Although initial reports seemed to support this hypothesis,^130,139^ systematic screening studies revealed that unreactive olefin-containing analogues, like rufomycin 21, exhibit superior antimycobacterial potency relative to their epoxide counterparts^140–142^ (Table 2), suggesting reactive epoxide-driven thioether conjugation was not necessary for antimycobacterial activity. Parallel studies found that the epoxide group in cyclomarin A was similarly insignificant on bioactivity.^143,144^ Yet, the stereochemistry of this group was found to be important, with analogues containing the *S*-configuration significantly outperforming the *R*-epimer in antimycobacterial potency^145^ (Table 2). This suggests that the orientation of the modified tryptophan, whose rotational freedom may be modulated by the steric properties of the functional handle, may be critical for appropriately orienting the peptide within the ClpC1 active site. This is further supported by complementary studies demonstrating that structural simplification of the olefin-bearing cyclomarin C peptide via removal of the tryptophan β-hydroxy group, yielding desoxycyclomarin C, reduced the peptide’s potency by 4-fold^146,147^ (Table 2). Conversely, modifying the unnatural β-methoxy phenylalanine residue with a para-nitro group enhances the potency of desoxycyclomarin C analogues by 8-fold^147^ (Table 2). Interestingly, this mirrors the critical role that the naturally occurring 3-nitro tyrosine residue plays in rufomycin bioactivity.^130,142^ Additionally, amino-4-hexenoic acid and amino-3,5-dimethylhexenoic acid residues act as essential hydrophobic anchors for rufomycin and cyclomarin, respectively, to the ClpC1 N-terminal domain.^148^ Analogues without this residue, like metamarin, show reduced inhibitory effects^149^ (Table 2). δ-hydroxy leucine, the final unnatural residue in cyclomarin, proved indispensable in additional SAR studies, where its modification significantly decreased the peptide’s activity.^128,148^ Similarly, rufomycins also possess an equally important 4-methyl-5-hydroxy-2-piperidinone moiety, which forms a unique cyclic hemiaminal ring that drastically improves the structural rigidity of this peptide.^150^ This leads to a lower entropic penalty for interaction with ClpC1 and, therefore, improved antimycobacterial activity^130^ (Table 2). Further potency enhancement of rufomycin 21 was achieved by replacing it with an even more conformationally rigid pyrrolidinone moiety.^151^

#### 3.3.2 Cyclic Depsipeptides

Depsipeptides represent another potent class of AMyPs with ClpC1 modulatory activity. Ecumicin is a well-studied representative of the class which features a 10-residue macrocycle connected to a 3-residue N-terminal tail via a threonine residue^152^ (Figure 5C). Later, two analogues with truncated tails, ohmyungsamycin (OMS) A and B, were identified as potent ClpC1 inhibitors using surface plasmon resonance experiments.^153^ Ecumicin and the OMS peptides were originally isolated as secondary metabolites from two actinomycete species, *Nonomuraea* and *Streptomyces*, respectively.^154,155^ Structural analysis revealed that ecumicin and OMS sequences engage a hydrophobic pocket on the ClpC1 N-terminus, with partial overlap with the rufomycin binding site^132^ (Figure 5B). Additional SAR studies on truncated OMS A analogues revealed that the flexible tail acts as a molecular anchor, stabilizing the complex through polar interactions with ClpC1^157^ and inducing an extended trimer complex that stimulates ATPase activity.^157^ Despite this activation, the peptide exerts antimycobacterial effects by sterically obstructing the ClpC1-ClpP1P2 interface, halting proteolytic degradation processes essential for mycobacterial survival^156^ (Figure 5D). Consequently, these scaffolds display submicromolar potencies against virulent *Mtb*, remain well tolerated by host cells, and are capable of clearing intracellular *Mtb*, with ecumicin showing potent inhibition of drug-resistant strains^152,158^ (Table 2). Furthermore, OMS A and ecumicin showed potent antimycobacterial clearance in drosophila and murine *in vivo* infection models, respectively.^152,158^

Finally, both ecumicin and OMS A possess two rare nonproteinogenic residues, N-methyl-4-methoxytryptophan and β-hydroxyphenylalanine, which are difficult to incorporate synthetically.^159^ Recent SAR investigations revealed that the removal of the β-hydroxy group significantly enhances potency, but deletion of the methoxy group weakens activity^160,161^ (Table 2). Additionally, ClpC1-ecumicin cocrystal structures implicate an N-methyl valine residue in the binding of a secondary ecumicin molecule at higher drug concentrations. SAR investigations revealed that this residue can be replaced with other hydrophobic alternatives like leucine, phenylalanine, or alanine without a significant loss of potency.^159,162^ Of these candidates, the alanine-substituted analogue, Ecu★ 4, achieved exceptional *in vitro* killing kinetics, completely eliminating *Mtb* within a week^163^ (Table 2). This was attributed to improved drug uptake or enhanced ClpC1 binding as a result of alanine’s smaller side chain. Furthermore, the ability of this AMyP candidate to synergize with established TB antibiotics like rifampicin, alongside its robust *in vivo* activity against *M. marinum* in zebrafish models, highlights its potential and justifies further preclinical evaluation.^163^

### 3.4 Targeting the Mycomembrane and Antibiotic Synergy

A key advantage of AMyPs, especially those that disrupt the mycobacterial cell envelope, is their potential to synergistically enhance the activity of small molecular antibiotics that otherwise poorly diffuse across the rigid mycomembrane. This approach is critical for tackling and resensitizing clinical *Mtb* strains resistant to first-line drugs like isoniazid and rifampicin. For instance, Hernandez et al. demonstrated that isoniazid, when dosed in combination with cationic hydrophobic cyclic AMyPs such as R_4_W_4_, show enhanced *Mtb* growth inhibition within *in vitro* granulomas^164^ (Table 2). Similarly, proteolytically stable D-conformers of linear cationic peptides like LAK120-A and LAK120-HP13 can potentiate isoniazid activity against MDR-TB clinical isolates through membrane permeabilization^165^ (Table 1). Notably, these peptides also bolster capreomycin, a second-line peptide antibiotic targeting ribosomal machinery.^166^ In this case, the enhancement stems not from increased membrane penetration, but from the peptides’ ability to mitigate the drug-induced membrane remodeling that confers resistance to the drug. Chemical conjugation of drugs to cell-penetrating peptides like Penetratin or Dhvar4 is another successful strategy utilized to improve the intracellular trafficking of antibiotics into lysosomal compartments.^167,168^ This targeted delivery into the intracellular niche of persister *Mtb* ensures higher localized drug concentrations and more complete bacterial eradication compared to physical mixtures.

### 3.5 AI-Driven Discovery and Optimization of AMyPs

Fueled by advances in the design of machine learning (ML) models, artificial intelligence (AI)-guided AMyP design has emerged as a promising avenue for rapid discovery and optimization of antimycobacterial agents. At its core, ML involves training computational algorithms to discern complex, nonlinear relationships between variables, which in the contexts discussed here, involves mapping peptide sequences and biophysical properties to their resulting mycobacterial inhibitory activity.^169^ Subsequently, these learned patterns are utilized for activity classification of known sequences, or the *de novo* generation of new candidates, with prediction accuracy typically correlated with the size and quality of the training data sets. This *in silico* screening approach can offer a more rapid, less costly, and labor-minimized method of exploring large sequence landscapes compared to standard peptide library chemical synthesis.^169^ Further, these methods have the potential to reveal novel mechanistic insights and drug design rationale that may have been previously hidden in biologic complexity. Such advantages have catalyzed the development of numerous ML models aimed at predicting general antimicrobial activity, each employing distinct strategies and achieving varying levels of predictive success.^170^ However, the utility of these generalized algorithms for identifying novel AMyPs is limited.^171^ Unlike Gram-positive or Gram-negative bacterial species, mycobacteria are defined by their distinct mycolic acid-rich envelope that presents a unique barrier to antibacterial peptides.^171,172^ Thus, ML models must be trained on mycobacteria-specific data sets to identify unique compositional requirements and ensure ML-based AMyP predictions are useful.^171^ To date, we are aware of 11 ML classifiers used for discovery of novel AMyPs, with each successive model demonstrating progressively improved prediction accuracy. To summarize the current state of the art for the reader, Table 3 lists all the reported ML-based AMyP prediction models along with their details and performance results. General insights from this early work have already identified early design rationale for AMyP efficacy, specifically highlighting an ideal hydrophobic sequence composition between 35%–45% and a net charge ranging from +2 to +4^173^.

**Table 3:** All AI-Based AMyP Prediction Models Reported in Literature

Ref	ML model name	Data sets employed	Final feature set *indicates feature selection application	Feature selection technique	Best model architecture	Performance (accuracy [data set]/AuROC [data set])
^173^	AntiTBPred	246 AMyPs + 246 inactive sequences (AntiTb_MD, AntiTb_RD)	AAC + N5C5-BP (Hybrid)	None	SVM	0.759 [MD]/0.83 [MD],0.785 [RD]/0.86 [RD]
^176^	AtbPpred	246 AMyPs + 246 inactive sequences (AntiTb_MD, AntiTb_RD)	AAC*, DPC*, CTF*, QSO*, GDPC*, GTPC*, N5C5-BP*, CTD*, AAI*	F-score + sequential forward search (SFS)	9 ERTs [Layer 1]; ERT meta-classifier [Layer 2]	0.894 [MD]/0.934 [MD],0.851 [RD]/0.899 [RD]
^181^	iAntiTB	246 AMyPs + 246 inactive sequences (AntiTb_MD, AntiTb_RD)	AAI + BP + DPC + TPC (Hybrid)	None	RF + SVM [Layer 1]; Linear regression meta-classifier [Layer 2]	0.808 [MD]/0.896 [MD],0.852 [RD]/0.946 [RD]
^178^	iATP	246 AMyPs + 246 inactive sequences (AntiTb_MD, AntiTb_RD)	g-gap DPC*	Incremental feature selection (IFS)	SVM	0.777 [MD]/0.85 [MD], 0.909 [RD]/0.96 [RD]
^180^	-	246 AMyPs + 246 inactive sequences (AntiTb_MD, AntiTb_RD)	(AAC + DPC + BP + Moran + CTD + PAAC + CTF + QSO + AAI + GDPC + GTPC)*	Divergence measure	Vote-based	0.878 [MD]/0.922 [MD],0.929 [RD]/0.914 [RD]
^175^	iAtbP-Hyb-EnC	246 AMyPs + 246 inactive sequences (AntiTb_MD, AntiTb_RD)	KSAAP + CPP + OHE (Hybrid)	None	RF + SVM + KNN + PNN + FKNN [Layer 1]; Genetic Algorithm (GA)-based meta-classifier [Layer 2]	0.927 [MD]/NA, 0.926 [RD]/NA
^174^	ATPfinder	262 AMyPs + 815 inactive sequences (Updated AntiTb_MD+RD)	AAC + PHYC + PAAC + DPC + CKSAAGP	None	Deep Forest	0.893/0.920
 ^179^	Hyb_SEnc	246 AMyPs + 246 inactive sequences (AntiTb_MD, AntiTb_RD)	(APAAC + DPC + ASDC + PAAC + QSO)*	Recursive feature elimination (RFE)	ERT + RF [Layer 1]; Logistic regression meta-classifier [Layer 2]	0.947 [MD]/NA, 0.957 [RD]/NA
^182^	LIMITS	110 Sequences (AntiTBPdb database and Ramon-Garcia et al.)	13 Distinct physiochemical properties	None	RF with iterative GA-based sequence refinement	0.94/0.956
 ^172^	-	80 Sequences (DBAASP database)	8 Distinct physiochemical properties	None	RF	0.80/NA
^177^	-	205 AMyPs + 235 inactive sequences (AntiTBPdb, GRAMP, DBAASP, DRAMP and APD databases)	OHE	None	LSTM with frozen encoder	0.905/0.969

Of particular importance to ML prediction accuracy is a well-curated training and validation data set. Despite the availability of diverse antimycobacterial sequences, curation is sporadic and inconsistent experimental protocols make standardization a major challenge.^173^ To address this issue, Usmani et al. compiled 542 unique, experimentally validated, AMyPs and published the resource in the AntiTbPdb online database^173^ (https://webs.iiitd.edu.in/raghava/antitbpdb/). In addition to sequence composition and antimycobacterial potency, AntiTbPdb contains information on peptide origin (natural vs synthetic), structural data, chirality, and biophysical properties. From this repository, two subset libraries of linear peptides (5–61 standard residues) were developed, named AntiTb_MD and AntiTb_RD, and used to train and validate the first AMyP prediction ML model: AntiTbPred.^171^ To ensure unbiased learning, each library contained equal numbers of positive (AMyPs) and inactive sequences (n = 246). While the AMyP data set was common across both libraries, the inactive data set was derived from different sources. For AntiTb_MD, inactive sequences were obtained from the DBAASP (Database of Antimicrobial Activity and Structure of Peptides) library, while for AntiTb_RD, inactive peptide lists were composed of random sequences generated using the Swiss-Prot database. Yao et al. further expanded and refined the entries in both the positive and negative data sets to improve AntiTbPred’s ability to distinguish AMyPs from general, broad-spectrum antimicrobials.^174^ Redundant sequences were removed from the database using CD-HIT, and length distributions were matched across sets to further minimize bias.^171,174^ Due to their size, diversity and curation, these data sets have been adopted as the standard training and validation resources for ML-based AMyP discovery. To further enhance the utility of this important resource, as part of this review we have published updated entries for both AntiTb_MD and AntiTb_RD data sets based on the novel linear sequences discussed (Table 1).

Development and validation of ML prediction models consist of a training and testing phase, both of which require a corresponding data set. A common practice is to utilize 80% of the data set entries for model training, and the remaining 20% to test and independently evaluate model performance (Figure 6). This strategy prevents model overfitting which may arise due to memorization of training data by the ML algorithm.^175^ To optimize training performance, hyperparameters (user-defined variables that dictate model complexity) must be tuned for any ML classifier using techniques like Grid Search or Bayesian optimization.^176,177^ During this process, performance metrics are often calculated using averaged values from k-fold cross validation studies.^176^

**Figure 6:**
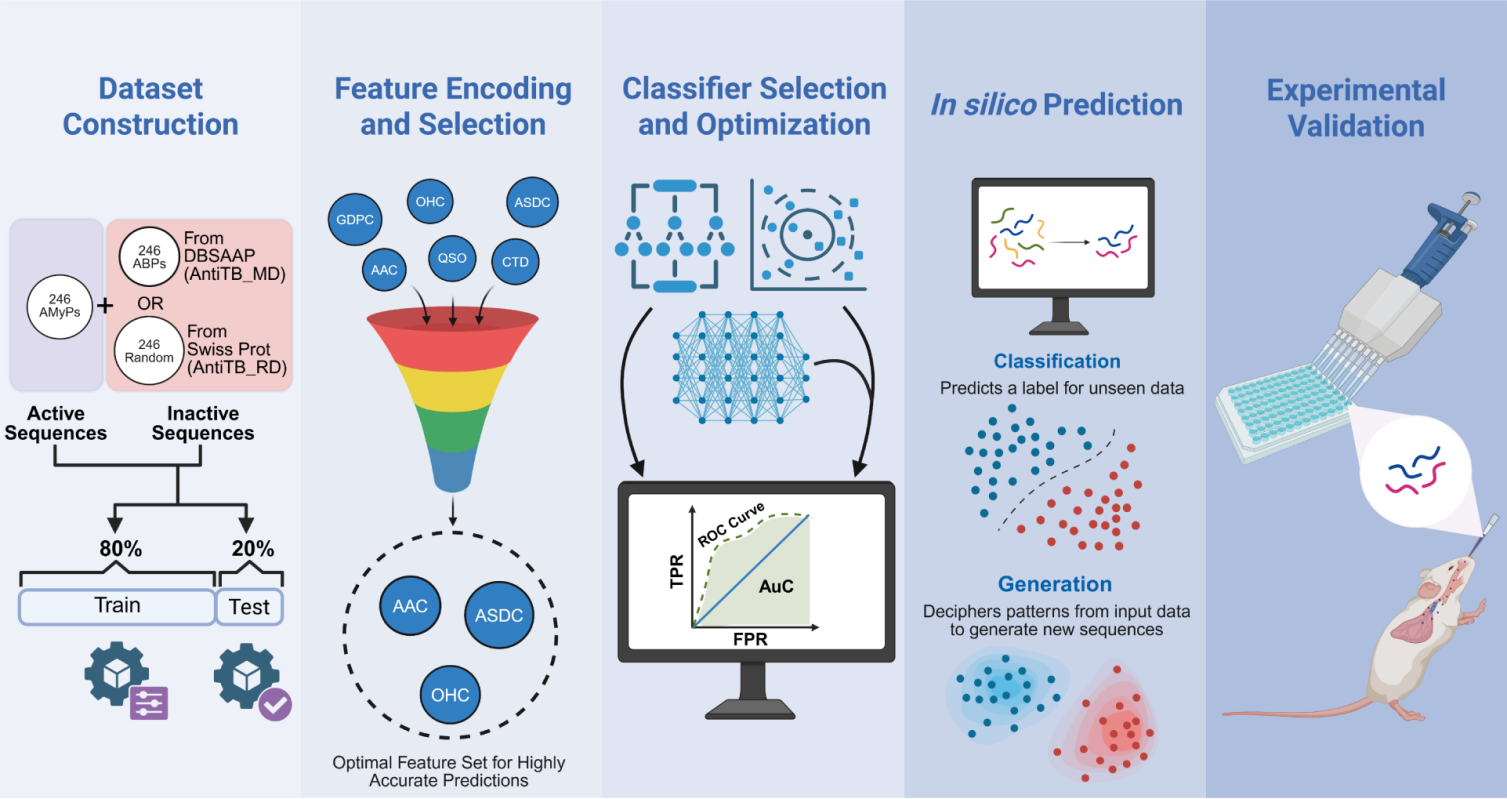
AI-driven workflow for AMyP discovery and optimization. ML model design and validation hinge on five core stages: curating balanced data sets, identifying optimal feature combinations, optimizing robust classifiers, producing experimentally tractable predictions, and validating *in silico* results through *in vitro* experimentation. ABP = Antibacterial peptides with no antimycobacterial activity; TPR = True Positive Rate; FPR = False Positive Rate; ROC = Receiver Operating Characteristic; AuC = Area under curve. Figure created with BioRender.com.

This method partitions the training data into “k” clusters, then cycles through the data so that every cluster is used once for evaluation while the other “k-1” clusters serve as the training set. Consequently, the training maximally covers the available data points in the set to allow as much variability as possible, ultimately improving the model’s generalizability and prediction accuracy.^176^ Implicit to the training process is the need to convert, or “encode”, peptide sequences into numerical features that can be employed by ML mathematical operations to learn data patterns. Encoding can be accomplished using sequence-based features, such as amino acid composition (AAC) or dipeptide composition (DPC), which quantify the frequencies of single residues or adjacent residue combinations, respectively.^171^ Longer-range relationships can also be captured using g-gap dipeptide composition and adaptive skip dipeptide composition (ASDC) features.^178,179^ Importantly, residue ordering within sequences can be mapped using one-hot encoding (OHC), in which each position is defined by a 20 dimensional binary vector representing each natural amino acid.^175^ Another method for sequence encoding involves the use of diverse global physicochemical properties like length, net charge, and hydrophobicity, which are important predictors of antimycobacterial potency.^172^ However, since these metrics fail to describe property distribution along the sequence, ML models can utilize features such as grouped dipeptide composition (GDPC) and conjoint triad feature (CTF) to map local and intermediate patterns.^176,180^ To resolve these arrangements at the residue level, amino acid indexes (AAIs) translate each residue into numerical physicochemical values.^176^ These values then serve as the basis for calculating pseudoamino acid composition (PAAC) and quasi-sequence-order (QSO) which quantify physicochemical correlations between residues at varying distances along the chain.^179,180^ Modern ML approaches typically utilize multiple feature encodings fused into a single hybrid vector to generate a holistic representation of training data. This strategy significantly improves prediction accuracy, as evidenced by several recent studies,^171,174^ but requires careful selection to eliminate irrelevant and redundant features.^180^ This is implemented by initially ranking features based on their relevance, using statistical measures like Fisher score, and then using iterative search algorithms such as Sequential Forward Search or Recursive Feature Elimination to identify the optimal feature subset that maximizes model performance.^176,179^

Given the unique challenges of synthesizing large peptide libraries, a key bottleneck in ML prediction of AMyP candidates is the limited size of training and validation data sets. Accordingly, early work favored Support Vector Machines (SVM) and Random Forest (RF) classifiers for AMyP prediction over neural networks, as they offer better prediction accuracy and generalization on limited data sets.^171,178,181^ The additional robustness of RF classifiers to noisy outliers has led to more sophisticated variants of this discovery strategy. For example, AtbPpred and ATPfinder utilize Extremely Randomized Trees (ERT) and Deep Forest classifiers, respectively, to employ an ensemble of decision trees that increased randomization, leading to improved performance relative to standard RF approaches using the AntiTb_MD and AntiTb_RD data sets.^174,176^ Important to AMyP discovery, where data set limitations remain a major hurdle to prediction accuracy, Deep Forest mimics the layered cascade architecture present in neural networks, while replacing neurons with RF decision workflows, to identify patterns from small data sets and minimize the risk of overfitting.^174^ Recent studies have additionally adopted ensemble learning, where decisions are jointly made by diverse classifiers to reduce individual model errors through collective assessment. This can be achieved either through simple averaging^171^ or using advanced techniques like stacking.^179^ Stacking employs a secondary model, known as a meta-classifier, to learn how to best combine the outputs from base classifiers. Depending on the complexity of data integration required, this meta-classifier can either be simple linear/logistic regression,^179,181^ or more complex algorithms like ERT.^176^

Another strategy that has yielded potent AMyP candidates under resource-scarce condition is coupling ML approaches with candidate optimization techniques that enable navigation of a complex, nonlinear parameter space. For example, our group has demonstrated that ML models trained on a limited, high-quality TB-specific data set can be effectively coupled with a genetic algorithm (GA) to discover highly potent and novel anti-TB candidates.^182^ This approach, referred to as Lead Informed Machine Interrogation of Therapeutic Sequences (LIMITS), was designed to leverage limited data sets (∼100 peptides in our first demonstration) to uncover potent, selective, and safe AMyPs by informing selection through lead candidate mutational scanning. Experimental validation showed that predicted sequences using LIMITS had nearly an order of magnitude improvement in potency, selectivity, and safety, relative to the initial AMyP template, highlighting the potential of hybrid approaches to enable discovery under data-limited conditions.^182^

Moving beyond AMyP classification, the latest ML models are deploying deep generative algorithms to discover *de novo* sequences. For example, Wang et al. used long short-term memory (LSTM)-based ML models, trained on a limited anti-TB peptide data set, to successfully generate multiple novel antimicrobial sequences.^177^ Because LSTMs excel at deciphering long-range patterns in sequential biological data (like DNA and proteins), they are increasingly integrated into multimodel pipelines to mine broad-spectrum antimicrobial peptides. Ma et al. designed a unified approach involving multiple natural language processing models (including LSTM) that work in conjunction with metaproteomics and correlational network analysis to discover potent sequences from massive metagenomic data sets of the human gut microbiome.^183^ As the gut is routinely exposed to, but rarely colonized by, environmental mycobacteria, it potentially serves as a rich source for deep learning-driven AMyP bioprospecting. This strategy can be readily adapted to other environments, such as soil and aquatic microbiomes as well. Finally, as demonstrated by Das et al., these generative pipelines can be coupled with high throughput molecular dynamics simulations to accurately screen large peptide data sets *in silico* for optimal physicochemical traits, such as enhanced mycomembrane interaction and minimal hemolysis.^184^ Ultimately, these deep learning approaches represent the frontier of AI-guided AMyPs discovery.

## 4 Conclusions and Future Directions

Advances in peptide synthesis, high throughput compound screening, and AI-guided sequence design has led to a proliferation of novel antimicrobial peptide scaffolds, with several clinically meaningful AMyPs recently reported. Despite this progress, several barriers remain to both compound discovery and clinical translation of novel candidates. With respect to natural product isolation, the inability to cultivate many of the environmental microbes that produce potential AMyP candidates limits the collection of biologic material necessary for screening efforts.^185^ Recent innovations in bacteriological cultivation, such as the isolation chip (iChip), that sequester previously unculturable microbes within semipermeable chambers are now offering new opportunities to overcome this limitation.^186^ Notably, this platform enabled the identification of a potent ClpC1 inhibitor, lassomycin, and the two AMyPs streptomycobactin and kitamycobactin.^185^ Complementary strategies, such as nutrient stress-induced metabolite production, will further expand access to otherwise transient antimicrobial expression pathways.^57^ Building on this, future efforts may consider the use of mycobacterial homogenates to stimulate specific immune responses that produce host defense AMyPs. In parallel, Chu et al. circumvented the need for cultivation entirely by devising a novel bioinformatics-guided synthetic strategy.^187^ This approach leverages established algorithms to mine bacterial genomes for cryptic NRPS gene clusters, predicting peptide sequences that are then chemically synthesized and cyclized according to structural trends observed in known NRPS-derived natural products.^187^ Collectively, these *in silico* and *in vitro* innovations promise to significantly broaden the pool of antimycobacterial scaffolds available from natural biorepositories.

As with other antimicrobial peptides, the translation of AMyPs often requires balancing potency with host toxicity, given the mechanistic links between membrane activity and cytolytic effects. For example, chemical modification of gramicidin S to attenuate its hemolytic activity resulted in a concomitant reduction in antimicrobial potency.^188^ Nevertheless, careful structure–activity studies can identify narrow opportunities for pharmacologic optimization. Pal et al. demonstrated that modifying the β-turn residues of gramicidin S to induce a distorted turn structure led to a significant enhancement in antimycobacterial potency, while reducing hemolytic activity.^189^ Other approaches for minimizing peptide toxicity, while preserving activity, often involve disulfide-mediated cyclization or hydrocarbon stapling.^190,191^ However, these strategies can require incorporation of nonproteogenic residues, which presents a significant barrier to biofermentative production and complicates large scale synthesis, underscoring the practical constraints that must be counterbalanced during AMyP therapeutic development. To navigate these complex physical and synthetic trade-offs, recent studies have reported sophisticated AI-driven web tools that allow *in silico* predictions of peptide toxicity (e.g., ToxTeller), hemolytic potential (e.g., HemoPI2), pharmacokinetic parameters (e.g., pepADMET) and membrane permeability (e.g., CPMP). Incorporation of these tools early in the AMyP development pipeline will further speed up candidate selection by triaging leads that may have toxicity or pharmacokinetic liabilities.^192^

Yet, despite the rapid progress that has already been made, AI-driven AMyP discovery is still in its infancy and remains limited by the availability of large training data sets. However, advances in spot array synthesis and high-throughput screening technologies hold promise to generate large peptide libraries that may address this barrier.^193^ These screening platforms can also uncover new SAR by exploring chemically diverse sequence spaces, including mixed-chirality and other non-natural architectures. For instance, rapid screening of 125,000 random sequences identified six mixed-chirality peptides that showed potent activity against multiple *M. abscessus* morphotypes.^194^ Data derived from such large-scale experiments are essential to evolve current ML-based AMyP predictive capabilities, enabling algorithms to move beyond natural linear sequences and toward accurate modeling of more complex peptide classes. In particular, extending predictive capacity to cyclic peptides represents a critical next frontier for AMyP discovery, as well as antimicrobial peptide research more broadly. Ultimately, continued methodological advances will allow ML models not only to identify membrane-active sequences, but also to support mechanism-guided rational design targeting specific metabolic and virulence pathways in mycobacteria.
